# Folk theories of gender and anti-transgender attitudes: Gender differences and policy preferences

**DOI:** 10.1371/journal.pone.0226967

**Published:** 2019-12-30

**Authors:** Mostafa Salari Rad, Crystal Shackleford, Kelli Ann Lee, Kate Jassin, Jeremy Ginges

**Affiliations:** 1 Kahneman-Treisman Center for Behavioral Science & Public Policy, Woodrow Wilson School of Public and International Affairs, Princeton University, Princeton, New Jersey, United States of America; 2 Department of Psychology, Princeton University, Princeton, New Jersey, United States of America; 3 Department of Psychology, New School for Social Research, The New School, New York City, New York, United States of America; Pontificia Universidade Catolica do Rio Grande do Sul, BRAZIL

## Abstract

Transgender rights and discrimination against transgender people are growing public policy issues. Theorizing from social, cognitive, and evolutionary psychology suggests that beyond attitudes, discrimination against transgender people may derive from folk theories about what gender is and where it comes from. Transgender identity is met with hostility, in part, because it poses a challenge to the lay view that gender is determined at birth, and based on observable physical and behavioral characteristics. Here, in two pre-registered studies (N = 1323), we asked American adults to indicate the gender of a transgender target who either altered their biology through surgical interventions or altered their outward appearance: to what extent is it their birth-assigned gender or their self-identified gender? Responses correlate strongly with affect toward transgender people, measured by feeling thermometers, yet predict views on transgender people’s right to use their preferred bathrooms above and beyond feelings. Compared to male participants, female participants judge the person’s gender more in line with the self-identified gender than the birth-assigned gender. This is consistent with social and psychological theories that posit high status (e.g., men) and low status (e.g., women) members of social classification systems view group hierarchies in more and less essentialist ways respectively. Gender differences in gender category beliefs decrease with religiosity and conservatism, and are smaller in higher age groups. These results suggest that folk theories of gender, or beliefs about what gender is and how it is determined have a unique role in how transgender people are viewed and treated. Moreover, as evident by the demographic variability of gender category beliefs, folk theories are shaped by social and cultural forces and are amenable to interventions. They offer an alternative pathway to measure policy support and possibly change attitude toward transgender people.

## Introduction

Gender is a primary way of classifying humans and is associated with numerous economic, social, and health outcomes. Perhaps no other category spans human cultures with more impact throughout life. The ubiquity of this category in its binary form, along with folk theories of what makes someone a man or a woman, or male or female, may explain why transgender identity is often met with discrimination and hostility [1–6]. Gender could be viewed as determined at birth, discernible based on some observable characteristics, and generally ascribed, or it could be viewed as a self-described identity that concerns an individual's sense of self and life history [7–9]. Transgender identity and its acceptance in mainstream society could pose a challenge to the former view. Thus, to the extent that people believe that the 'true gender' of transgender individuals is the gender they were assigned at birth, we can expect them to hold negative affect toward transgender people [10,11]. We make two additional predictions based on this hypothesis. First, folk theories of gender are likely to capture unique variance in views toward transgender-related policies, above and beyond affect (i.e., feelings of warmth) toward transgender people. Secondly, because theories of gender are influenced by the perceiver’s social experiences and motivations [12–15], female and male perceivers are likely to differ systematically at categorizing transgender people in terms of gender. We present our arguments along with the supporting literature below (Our focus here is on the gender concepts of cisgender, heterosexual men and women).

Several lines of research motivate the link between folk beliefs about gender and attitudes toward transgender people. Social psychological research posits that gender is a product of social, cultural, and situational forces interacting with biological and physical attributes of men and women [16,17]. Ecological and biological constraints on behavior define specific roles and expectations for different gender groups within a culture, and foster the belief that men and women essentially differ in disposition and belong to separate categories. Transgender people could face hostility because by moving between spaces, they could be perceived as threatening or undermining a ‘natural’ system of social and biological organization [1,18]. Research on perception and categorization shows that negative evaluations of individuals and groups can be caused by ambiguity and classification difficulty [19–22], as well as role incongruence and counter-normative behaviors [23]. By identifying with a gender other than their birth-assigned gender, transgender people could be seen as imposing a cognitive tax on lay perceivers and thereby becoming targets of antipathy. There is also an evolutionary perspective, which confers an adaptive value to gender-based categorization [24]. Goals of survival and reproduction could have rendered people sensitive to cultural cues that distinguish between males and females [25]. In lay view, transgender people might seem deviant and be evaluated negatively because they seem to distort signals when they are viewed as trying to 'pass' as a gender other than their birth-assigned gender [26,27]. Together, these perspectives suggest that gender category beliefs, specifically the folk beliefs regarding how gender is determined, are associated with how transgender people are viewed and treated.

Folk theories of gender are the causal explanations and intuitive reasoning that underlie lay classification of individuals into gender groups [28–33]. The overarching system of beliefs that designates a person as a man or a woman is undergoing a cultural and scientific transformation to recognize a spectrum of gender with a primary role for the self in determining one’s gender [8,34,35]. As discussed above, this could face resistance and result in aversion towards transgender people because they seem to pose a threat to the view that gender is determined at birth and based solely on physical characteristics. In other words, gender category beliefs and affect toward transgender people are likely to covary. However, we expect folk theories to capture unique variance in policy preferences toward transgender people for two reasons.

Firstly, affect and belief could be associated with different forms of discrimination. Affect is strongly associated with interpersonal discriminatory behavior, whereas beliefs are part of one’s worldview and thus linked to more abstract forms of discrimination such as public policy attitudes [36]. Folk theories of gender may uniquely predict attitudes towards more abstract, non-personal, and ideological policy questions, such as whether people should have the right to use the bathroom that aligns with their self-described gender. In contrast, feelings toward transgender people, might for example, better predict whether a person will engage in discriminatory behaviors, such as actively protest or seek to prevent a transgender person from using their bathroom of choice. Other research shows that policy preferences can be more affect-driven or cognition-driven depending on contextual and demographic variables [37]. Accordingly, folk beliefs and affect could each explain unique variance in policy preferences toward transgender people.

A second reason why the distinction between affect and beliefs might contribute separately to policy preferences is that some people may refrain from expressing felt negative affect towards other groups [38,39], but they might see it as fair and legitimate to have a certain perspective about what gender is and how it is determined. In the context of anti-immigration attitudes for example, social norms against prejudice influence the relationship between policy preferences and attitudes [40]. People might understate their dislike of transgender people and reconcile their preference for discriminatory policy by appealing to their theories of gender and perceived consequences of altering policies. Others might harbor negative feelings toward transgender people while accepting that gender is, at least in part, self-declared and thus oppose discriminatory policy positions. For example, [41] showed that in Germany, proximity to immigrants is associated with anti-immigrant feelings but does not affect policy preferences. In sum, people might not report their true affect, and policy positions are not entirely determined by affect. In these cases, beliefs might capture some variability in policy preferences that feelings might miss, and this can assist us in measuring policy support and designing interventions [42].

We also predict theories of gender to differ systematically between male and female participants. Prior research has shown that folk beliefs about category membership are dynamic and shift as a function of the socio-cultural environment and across the lifespan [13–15]. They are also motivated by the perceived consequences of differing beliefs for the self and the ingroup [12,43,44]. For men and women, gender has divergent ramifications and they are thus likely to hold variant folk beliefs [45–47], specifically differing in the degree to which they view gender as ascribed or self-described [48,49]. This disparity is predicted from a social psychological view [50–53]. Gender category beliefs are influenced by one’s experience of the gender binary and gender roles, which correspond with asymmetries in status and relative positions on the gender hierarchy [9,52,54]. Low-status groups perceive hierarchies as more permeable and fluid, whereas high-status groups perceive them as more rigid and stable. This is especially the case when a change in the hierarchy is perceived to benefit or harm one’s ingroup [43,44]. Viewing a person's gender as what they say it is and not determined by others at birth could seemingly destabilize the conventional gender binary system that fundamentally organizes social relations. This impacts males and females differently. We thus expect females to view gender identity as more self-declared and less determined at birth than males.

In measuring folk theories of a category, we adopt an approach employed by psychologists and anthropologists [55–57]. Broadly, we present participants with a stimulus and ask them to categorize it. By systematically varying the stimulus on features of interest, we can infer the role or weight of that feature in the subject’s theory of that category. Here and in other research on folk theories of nationality and religions, we take this approach but with some modifications [58,59]. Specifically, the stimulus (e.g., a person) moves from one category to another via a specific procedure (e.g., adoption, surgery), and participants use continuous scales to indicate the extent to which it belongs to either category. Thus, the movement between categories is a feature of study (i.e., What procedure was taken? In which direction did it occur? etc.), and we can tease apart the degree to which membership in each category can be gained or lost. This is particularly informative when the participants identify as a member of the categories in question.

We find this approach to be more suitable to study folk theories for several reasons. Firstly, it does not require participants to explicitly report their beliefs about groups of people, which could be a sensitive topic and prone to self-presentation bias. In a sense, responses obtained this way are likely to reflect revealed beliefs rather than stated beliefs, which is particularly important in policy preferences. For example, [60] showed that Swiss natives’ vote on naturalization rights varies based on the immigrants’ origins, but in public opinion surveys these differences are absent or reversed. Using vignettes, we provide a context to participants and ask them to make multiple judgments about a person, instead of asking them to express their agreement with statements that are prone to desirability biases (e.g., ‘*Transvestites are people who gain pleasure from cross-dressing*’, [6]).

Two studies deserve specific attention here. One is the seminal survey [10] that shows how anti-transgender prejudice, measured by the feeling thermometer scores, varies as a function of demographics variables of gender, race, education, age, ideology, religiosity, as well as region and type of residence in a large sample of US adults [10]. The authors also look at binary conceptions of gender, using a single item (‘*These days there is not enough respect for the natural divisions between the sexes*.’). While they find a significant correlation between gender binary beliefs and feeling thermometer scores (*r*(2281) = -0.26, *p* < 0.001), they do not find men and women to significantly differ in such beliefs. We use the same feeling thermometer task to measure affect toward transgender people. But we improve measurement of gender beliefs using the folk theories approach outlined above. This paper is noteworthy for its large sample and sophisticated analysis but its primary focus is on determinants of affect toward transgender people and does not measure policy preferences.

Another article is [6] that focuses specifically on transgender civil rights, and explores its variability as a function of multiple factors: beliefs about gender, beliefs about transsexuality, similarity of different gender groups, several prejudice scales, contact with gender and sexual minorities, and several demographic variables. This paper uses a series of scales (80+ items) and does not measure affect toward transgender people.

We extend prior work by measuring affect toward transgender people, folk theories of gender, and policy preferences about bathroom use. We measure affect using the thermometer scales adopted in the large-scale studies by [5] and [10]. Moreover, we measure folk theories of gender using a technique that does not rely on self-reporting agreement with abstract statements regarding gender, but instead gives participants a more realistic context by asking them to make direct judgments about the gender of transgender targets. It is also much simpler, which facilitates cross-cultural research because it does not require translation and validation of scales that list a multitude of items.

## Methods

The research is approved by the Institutional Review Board at the New School for Social Research (New School IRB# 2016–1041). To test our predictions, we begin by measuring variability in folk theories of gender as a function of type and direction of gender transition procedures, as well as the perceiver’s gender. We then examine the relationship between folk beliefs and attitudes toward transgender people, and compare how the two perform in predicting preferences for transgender restroom policy. We further examine gender variability in folk beliefs as a function of age, political ideology, religion, race, and state of residence. We conducted two studies with similar procedures.

Participants initially read a consent form, approved by the New School IRB (#2018–1041). They were then asked to read hypothetical scenarios, describing a character who was labelled and raised as one gender at birth, but who identified as a different gender and took steps to align either their appearance or biology with what they believe to be their true gender. The procedure the character used varied between participants. It either involved changes of gender in name, appearance and clothing (‘non-biological’), or also included surgery and hormone therapy (‘biological’). We also varied the direction of the transition (i.e., assigned at birth male to female, assigned at birth female to male). An example of the non-biological, male-to-female transition was:

‘*Jack was born with the sexual characteristics of a male and was raised as a boy. As Jack grew up, Jack began to identify more and more as a woman. When Jack became an adult, Jack stopped going by their legal name, and started answering to the name of “Jill.” Jill grew long hair, likes to wear clothes that are thought of as feminine, and wears lipstick to work*.’

An example of the biological transition scenario in the female-to-male direction was:

‘*Jill was born with the sexual characteristics of a female and was raised as a girl. As Jill grew up, Jill began to identify more and more as a man. When Jill became an adult, Jill stopped going by their legal name, and started answering to the name of “Jack.” Jack’s hair is now cut very short, and Jack likes to wear clothes that are thought of as masculine. After much thought and counselling, Jack decided to undergo hormone therapy, surgical removal of breasts, and surgical construction of a penis*.’

We used proper names to avoid potential confusion over the use of changing gender pronouns (See Section B in S1 File for details of study instruments). Each participant was randomly assigned to read one of the four scenarios. All participants responded to the same 4 questions in random order on a 0–100 scale: ‘*To what extent do you think Jill* [*Jack*] is a *male* [*female*/*man*/*woman*]?’ Asking about both genders enabled us to measure the weights of the assigned-at-birth and the self-identified genders in gender category beliefs. We also asked participants to briefly explain their answers in a text box. This probe nudged the subject to think about the scenarios and if need be, modify their responses (See Section H in S1 File for a content analysis of these explanations).

Note that depending on the direction of transition, the same question could ask about either the assigned-at-birth or the self-identified genders. For example, in the male-assigned to the female-identified scenario, asking ‘*to what extent is Jack a man*’ measures the extent to which the target has the birth-assigned gender (i.e., to what extent is the birth-assigned gender ‘retained’?). In the female-assigned to male-identified scenario, the same question measures the degree to which the target has the self-identified gender (i.e., to what extent is the self-identified gender ‘acquired’?). As discussed in the introduction, this method could disentangle beliefs about stability and change in category membership. Here specifically, we can examine whether and how in lay view, gaining and losing membership in gender categories varies depending on direction and type of the transition scenarios, as well as the labels we used in the questions. We also use different sets of labels because there may be variation in the degree to which lay theories distinguish between male/female and man/woman labels. Thus, the four questions measuring gender category membership are grouped in two factors: one based on the gender in question and the direction of the scenario (Frame: assigned gender, identified gender) and one based on the Label: female/male, man/woman).

In sum, the study design was 2 (Transition: *biological*, *non-biological*) X 2 (Direction: *male to female*, *female to male*) X 2 (Frame: *assigned gender*, *identified gender*) X 2 (Label: *male*/*female*, *man*/*woman*). Type and direction of transition were between-subject factors, and frame and label were crossed within subject.

Following a distraction task, participants responded to “feeling thermometer” questions [10] about four groups (*men*, *women*, *transgender men*, and *transgender women*) in random order on a 0–100 scale. The wording of the task was as follows:

*‘Using this scale from 0 to 100*, *please tell us your personal feelings toward each of the following groups*. *As you do this task*, *think of an imaginary thermometer*. *The warmer or more favorable you feel toward the group*, *the higher the number you should give it*. *The colder or less favorable you feel toward the group*, *the lower the number*. *If you feel neither warm nor cold toward the group*, *rate it 50*.’

*[*Men, Women, Transgender men, Transgender women*]*

Next, participants gave their views on the use of bathrooms by transgender men and transgender women that match their self-identified genders. Participants read the following:

‘*It would be wrong to allow a transgender man [woman] (a person who identifies as a man [woman] but was designated female [male] at birth) to use the men's restroom*.’

They could select one of two choices, ‘*Yes*, *it is wrong*’ or ‘*No*, *it is not wrong*’, coded as 0 and 1.

Lastly, participants indicated their sex (Female, Male, Other), age group (18–25, 26–34, 35–54, 55–64, 65+), political ideology (‘*Very liberal’* (1)—‘*Very conservative’* (7)), race (*White*, *Black*, *Hispanic*, *Asian*, *Native American*, *Pacific Islander*, *Other*), religion (*Christian*, *Muslim*, *Jewish*, *Hindu*, *Buddhist*, *Mormon*, *Other*, *Atheist*, *Agnostic*), and their state of residence.

## Results

In Study 1, pre-registered (osf.io/m7vhk), we recruited N = 623 participants (F = 329, *M*_*age*_ = 35) from the United States. The majority of participants was White (76.4%) and reported having some religion (54.5%). The sample is almost evenly distributed across the ideological spectrum (*M* = 3.44, SD = 1.74). For demographics, exclusions, and power analysis, Section 2 in S1 File.

We analyzed responses to the folk theories task using a 2 Transition (between: *biological*, *non-biological*) X 2 Direction (between: *male to female*, *female to male*) X Participant sex (between: *male*, *female*) X 2 Frame (within: *assigned gender*, *identified gender*) X 2 Label (within: *male*/*female*, *man*/*woman*) mixed ANOVA. Results showed a significant Transition Type X Frame interaction (*F*(1,619) = 19.580, *p* < 0.0001, *η*^2^_g_ = 0.026), a significant Participant sex X Frame interaction (*F*(1,619) = 32.202, *p* < 0.0001, *η*^2^_g_ = 0.043), and a significant within subject interaction of Label X Frame (*F*(1,619) = 9.364, *p* = 0.0023, *η*^2^_g_ = 0.0009).

The type of transition procedure, specifically whether it involved surgery and hormonal therapy, played a significant role in how participants viewed the character’s gender. Participants thought the self-identified gender was acquired more than the assigned-at-birth gender was retained in the biological scenario (*M*_*diff*_ = 16.7, *SE* = 4.09, *t*(619) = 4.085, *p* < 0.0001). That is, participants judged their true gender to be more in line with their self-identified gender than the gender they were assigned at birth. The reverse was true in the non-biological scenario where the assigned-at-birth gender was retained more than the self-identified gender was acquired (*M*_*diff*_ = 10.1, *SE* = 4.06, *t*(619) = 2.493, *p* = 0.0129). So, when transgender targets did not undergo medical interventions, participants judged their true gender to be more in line with their birth-assigned gender than their self-identified gender.

However, regardless of the type of transition procedure, female participants viewed the self-identified gender as more acquired than the assigned-at-birth gender was retained (*M*_*diff*_ = 19.7, *SE* = 3.95, *t*(619) = 4.986, *p* < 0.0001). For male participants, the assigned-at-birth gender was more retained than the self-identified gender was acquired (*M*_*diff*_ = 13.1, *SE* = 4.2, *t*(619) = 3.121, *p* = 0.0019). Lastly, the self-identified gender was acquired slightly more than the assigned-at-birth gender was retained when the labels ‘*man*’ and ‘*woman*’ were used (*M*_*diff*_ = 6.13, *SE* = 2.9, *t*(708) = 2.054, *p* = 0.0403). The two did not significantly differ when we used the labels ‘*male*’ and ‘*female*’, (*t* < 1, *p* > 0.8). For full ANOVA tables see Section C in S1 File (All contrasts are Bonferroni corrected).

We reversed each subject’s response in the retain frames and averaged them with their responses in the acquire frames to calculate an index of ‘Gender Category Beliefs’ or GCB (*α* = 0.95, *M* = 53.1%, *SD* = 37.5). The higher the GCB scores, the less the birth-assigned gender is retained and the more the self-described gender is acquired. In other words, the assigned and the identified genders carry less and more weight in one’s theory of gender respectively. About half of our sample (38.1%) fell on the extremes of this score. Specifically, GCB of 18.2% of participants was zero, meaning they viewed the character’s gender as fully in line with the assigned-at-birth gender; for 19.9% of participants, the person had the self-identified gender. The majority of participants assume some role for the self in determining gender, yet a sizable minority maintains gender to be solely designated at birth.

**Fig 1:**
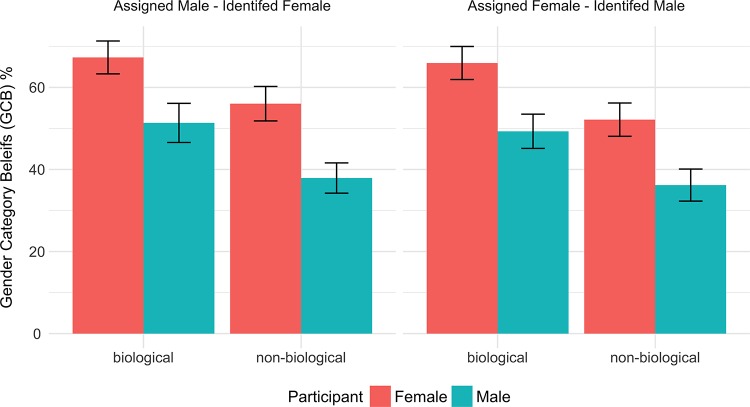
Gender category beliefs (GCB), calculated by averaging the ratings of the identified gender and reversed ratings of the assigned gender, split by transition type and direction, and participant gender. Gender is more identified than assigned in the eyes of female participants compared to male participants, regardless of the type or the direction of the transition procedure.

We conducted a 2 Transition (between: *biological*, *non-biological*) X 2 Direction (between: *male to female*, *female to male*) X Participant sex (between: *male*, *female*) ANOVA to predict GCB scores. Results showed a significant effect of Participant sex, (*F*(1,619) = 32.795, *η*^2^_g_ = 0.050, *p* < 0.0001), where GCB was higher in female participants than male participants (*M*_*diff*_ = 16.62%, 95%CI[10.9,22.3]) and it was higher in the biological scenario than in the non-biological scenario (*F*(1,619) = 19.670, *p* < 0.0001, *η*^2^_g_ = 0.030, *M*_*diff*_ = 13.4, 95%CI[7.7,19.1]). For both types and directions of transition procedures, female participants viewed the person more according to the self-identified gender than the birth-assigned gender, compared to male participants. This suggests that social and structural aspects of beliefs about gender are, in part, independent of the extent to which a person’s body parts match their self-identified gender. Moreover, participants responded to male-to-female and female-to-male transitions similarly (i.e., direction of transition: *F* < 1, *p* > 0.3), meaning responses were likely based on their beliefs about how gender is determined and to what extent transition between genders is possible, rather than specific beliefs about men or women, or ingroup and outgroup genders. Fig 1 shows a summary of these results. For complete ANOVA tables see Section C in S1 File.

**Fig 2:**
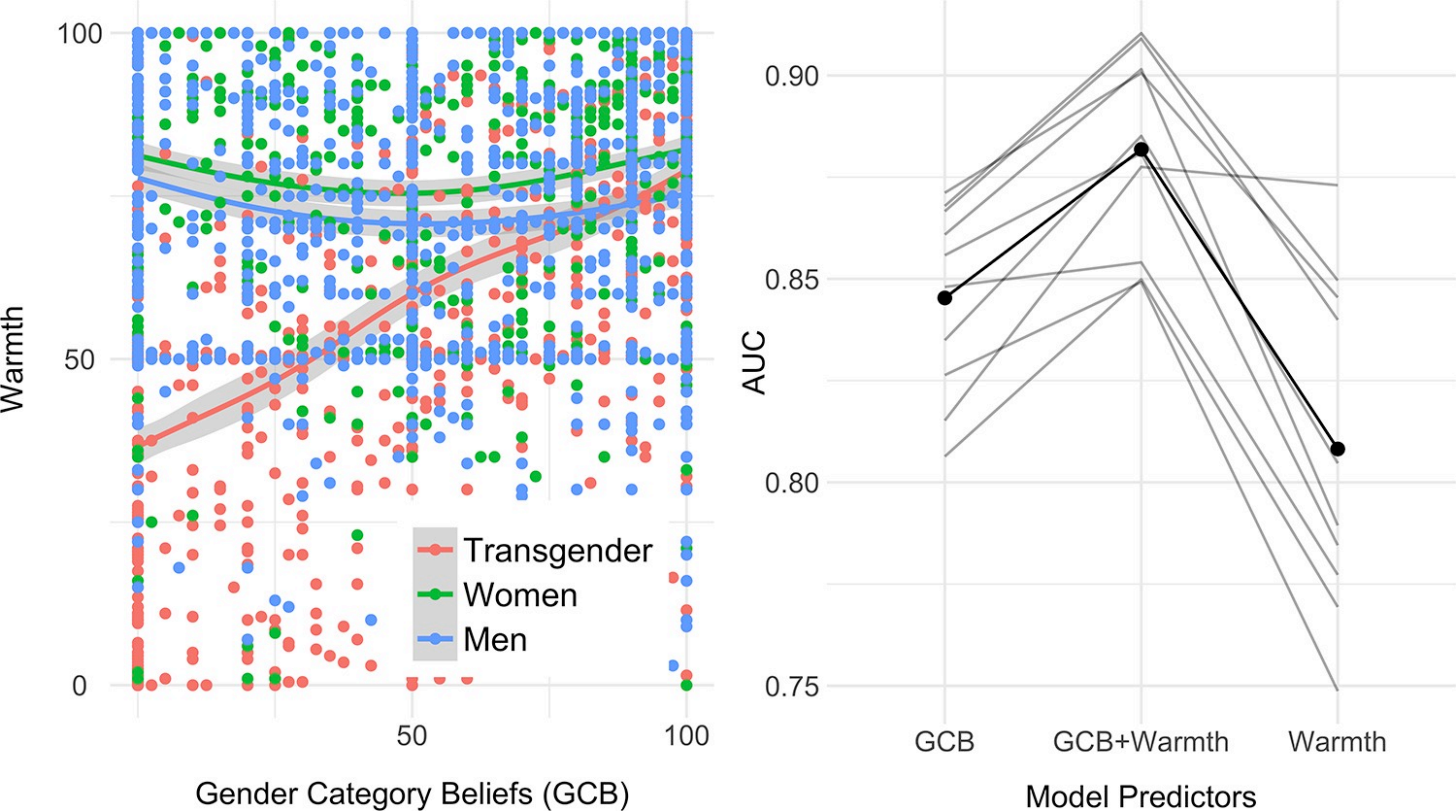
Panel on the left shows GCB scores and attitudes (i.e. warmth felt) toward transgender, men, and women in the pooled sample, with locally weighted regression curves. Higher GCB is associated with more warmth towards transgender people. Panel on the right plots area under the curve (AUC) of Receiver Operator Characteristic from 10-fold cross-validation performed on three models predicting bathroom choice preferences with Warmth, GCB, or Warmth+GCB. The grey lines are performances in each fold. The black line is mean model performance from the 10 folds. GCB is a better predictor of policy preferences than Warmth, but the best model includes both variables. See Sections E-F in S1 File for details of this analysis, comparisons with other models, and other analyses.

In the “feeling thermometer” scores, we find feelings of warmth or attitudes toward transgender men and transgender women to be highly correlated, (*α* = 0.95, *p* < 0.0001). We averaged them into a single score of feelings toward transgender people, (*M* = 59.4%, *SD* = 28.7). We then tested a linear mixed model to predict participants’ thermometer ratings with GCB scores, participant sex, and the target of the thermometer ratings (*Men*, *Women*, *Transgender people*). Results showed that GCB scores predicted feelings of warmth, but this relationship depended on who was the target of the thermometer task (*F*(2,1238) = 176.827, *p* < 0.0001). GCB correlated with attitudes toward transgender people, (*ρ* = 0.45, *p* < 0.0001) but not with attitudes toward men or women (*ρ*s < 0.1, *P*s > 0.1), for both male and female participants (*ρ*_women_ = 0.44 vs. *ρ*_men_ = 0.42, *P*s < 0.0001). This suggests that beliefs about the degree to which gender is assigned or self-identified are specifically related to hostility to transgender people rather than some general antipathy towards one gender group. See Fig 2A. We also find a significant main effect of participant sex. Female participants showed more positive affect in general than male participants, (*M*_*diff*_ = 5.06, *SE* = 1.47, *t*(619) = 3.455, *p* = 0.0006). This is consistent with prior work that shows women hold less negative attitudes toward transgender people [48,61]. See Section E in S1 File for distributions and full results.

We pre-registered and replicated these results in Study 2 with minor changes to the methods (N = 702, F = 352, *M*_*Age*_ = 37). We altered the wording of the dependent variable to make it more neutral by replacing the character’s chosen name with ‘*this individual*.’ Results replicate the findings from Study 1. A 2 Transition (between: *biological*, *non-biological*) X 2 Direction (between: *male to female*, *female to male*) X Participant sex (between: *male*, *female*) X 2 Frame (within: *assigned gender*, *identified gender*) X 2 Label (within: *male*/*female*, *man*/*woman*) mixed ANOVA model showed significant interactions between Transition Type X Frame (*F*(1,694) = 19.710, *p* < 0.0001, *η*^2^_g_ = 0.024), Participant sex X Frame (*F*(1,694) = 11.490, *p* < 0.0001, *η*^2^_g_ = 0.014), and a within subject Label X Frame interaction (*F*(1,694) = 15.197, *p* = 0.0001, *η*^2^_g_ = 0.0009). The self-identified gender was acquired more than the birth-assigned gender was retained in the eyes of the female participants than male participants, and in the biological transition than non-biological transition. Also, when using the labels ‘*man*’ and ‘*woman*’, the self-identified gender was acquired significantly more than the assigned-at-birth gender was retained (*M*_*diff*_ = 9.92, *SE* = 2.85, *t*(694) = 3.481, *p* = 0.0005), but not when the labels male and female were used, (*M*_*diff*_ = 5.16, *SE* = 2.85, *t*(694) = 1.812, *p* = 0.0704). As in Study 1, participants did not differentiate between male-to-female and female-to-male transitions (*F* < 1, *p* > 0.8).

We calculated a GCB score for each subject (*α* = 0.96, *M* = 53.7, *SD* = 37.4). An ANOVA model with 2 Transition (between: *biological*, *non-biological*) X 2 Direction (between: *male to female*, *female to male*) X Participant sex (between: *male*, *female*) ANOVA showed significant effects of Participant sex (*F*(1,694) = 11.447, *η*^2^_g_ = 0.016, *p* = 0.0007) and Transition type (*F*(1,694) = 19.593, *η*^2^_g_ = 0.027, *p* < 0.0001). GCB was higher in female participants than male participants (*M*_*diff*_ = 9.34, 95%CI[3.89,14.7]) and it was higher in the biological scenario than in the non-biological scenario (*M*_*diff*_ = 12.5, 95%CI[7.05,17.94]). See Section C in S1 File for full results.

Next, a linear mixed model predicting thermometer rating for different groups using GCB, participant sex, and target group showed the moderating effect of target group on the relationship between GCB and feelings of warmth (*F*(2,1394) = 223.723, *p* < 0.0001). As in Study 1, GCB correlated with attitudes toward transgender people, (*ρ* = 0.58, *p* < 0.0001) but not with attitudes toward men or women (*ρ*s < 0.5, *P*s > 0.11), for both male and female participants (*ρ*_women_ = 0.58 vs. *ρ*_men_ = 0.53, *P*s < 0.0001). Female participants showed more positive affect in general than male participants, (*M*_*diff*_ = 3.13, *SE* = 1.26, *t*(697) = 2.478, *p* = 0.0135). See Section E in S1 File for distributions, full results, and plots.

In the pooled sample (N = 1323), we find no effect of study (*p* > 0.13) but find similar results to Study 1 and Study 2 (Participant sex X Frame: *F*(1,1313) = 40.409, *p* < 0.0001, *η*^2^_g_ = 0.026; Transition type X Frame: *F*(1,1313) = 41.150, *p* < 0.0001, ^2^_g_ = 0.026; Frame X Label: *F*(1,1313) = 28.616, *p* = 0.0011, *η*^2^_g_ = 0.001). The GCB scores were higher in female participants than male participants (*M*_*diff*_ = 12.6, *SE* = 2, *t*(1321) = 6.312, *p* < 0.0001), and in the biological transition than in the non-biological transition, (*M*_*diff*_ = 13, *SE* = 2, *t*(1321) = 6.531, *p* < 0.0001).

**Fig 3:**
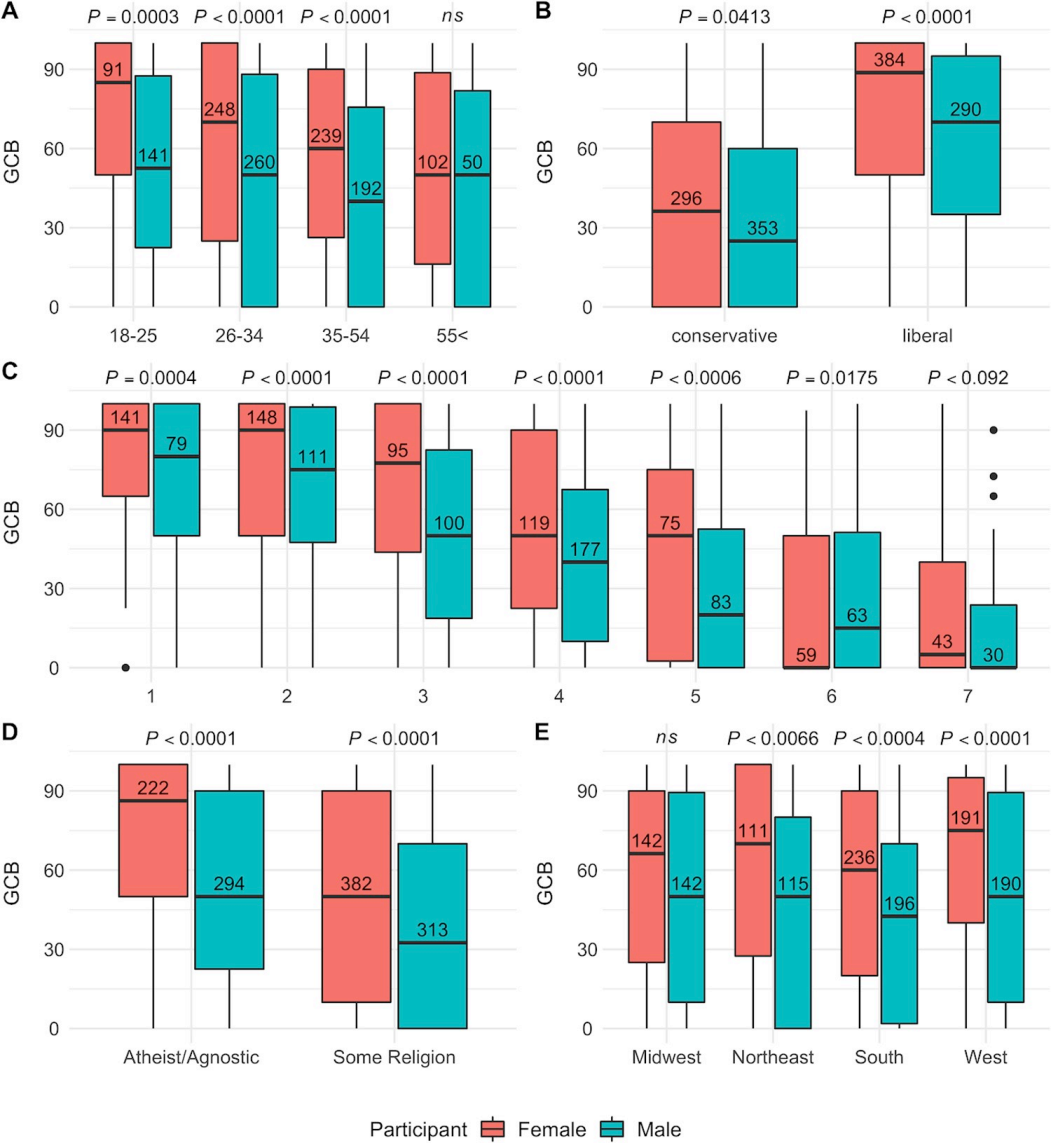
Gender category beliefs (GCB) as a function of participant sex and other demographic variables. Panel A shows how GCB in different age groups. Panel B shows the dichotomized political ideology variable (1–3: Liberal, 4–7: Conservative). Panel B shows variability in GCB as a function of the continuous political ideology scale (1: Very Liberal—7: Very Conservative). Panel D plots GCB in atheist/agnostic people next to people who reported as having a religion. Panel E shows GCB in different regions. Numbers on the box plots are the number of participants. P-values are obtained via Bonferroni-adjusted post hoc tests. See Section D in S1 File for details. Overall, female participants view the self-identified gender as more acquired and the birth-assigned gender as less retained than male participants, regardless of the process or direction of the gender alignment procedure. However, this difference is larger in the subsample that is liberal, young, non-religious, and not from the Midwestern United States.

We further examined GCB variability by age, political ideology, state of residence, and all of these variables combined. An ANOVA model predicting GCB with age (combined 55–65 year-olds and 65+ and year-olds into one group) and participant sex showed main effects of age (*F*(3, 1315) = 3.670, *p* = 0.0138, *η*^2^_g_ = 0.013) and participant sex (*F*(1, 1315) = 3.670, *p* < 0.0001, *η*^2^_g_ = 0.033) but no interaction (*p* > 0.3). Post Hoc tests with Bonferroni correction showed the female participants had higher GCB than male subject in age groups of 18–25 (*M*_*diff*_ = 17.76, *SE* = 4.95, *t*(1315) = 3.590, *p* = 0.0003), 26–34 (*M*_*diff*_ = 13.39, *SE* = 3.26, *t*(1315) = 4.103, *p* < 0.0001), 35–54 (*M*_*diff*_ = 15.44, *SE* = 3.56, *t*(1315) = 4.332, *p* < 0.0001), but at over 55, the two groups were not significantly different in GCB (*p* > 0.4). Fig 3A summarizes this result. For full results and pairwise comparisons see Section D in S1 File.

A linear model predicting GCB scores with political ideology (*M* = 3.43, *SD* = 1.76, *Median* = 3) and participant sex showed that GCB scores decrease as we move to the right of the ideological spectrum (*b* = -9.87 (*SE* = 0.51), *t* = 19.059, *p* < 0.0001), and while female participants have higher GCB than male participants, (*b* = 5.90 (*SE* = 2.00), *t* = 2.946, *p* = 0.0032), the interaction is not significant, (*t* < 1, *p* > 0.4). However, post hoc tests with Bonferroni correction showed that with increased conservatism, the gender difference in GCB becomes smaller. Fig 3C shows this pattern. We calculated a dichotomous political ideology variable based on the median split of the continuous political ideology variable (*Liberal* (1–3): N = 674; *Conservative* (4–7): N = 649). A 2 ideology (Liberal, Conservative) X 2 participant sex (Female, Male) ANOVA showed significant main effects of political ideology (*F*(1,1319) = 267.57, *p* < 0.0001, *η*^2^_g_ = 0.168) and participant sex, (*F*(1,1319) = 23.666, *p* < 0.0001, *η*^2^_g_ = 0.017), and no interaction, (*p* = 0.0535). Post hoc tests showed that female participants had higher GCB than males in both groups but the difference in liberals (*M*_*diff*_ = 12.64, *SE* = 2.62, *t*(1319) = 4.820, *p* < 0.0001) was over twice the size of the difference in conservatives (*M*_*diff*_ = 5.43, *SE* = 2.66, *t*(1319) = 2.042, *p* = 0.2480). The largest difference in GCB is between liberal females and conservative males, (*M*_*diff*_ = 39.47, *SE* = 2.49, *t*(1319) = 15.878, *p* < 0.0001). See Fig 3B. For other contrasts, see Section D in S1 File.

For religion, we categorized those who identified with a religion (N = 695) in one group, those who explicitly identified as Atheist/Agnostic (N = 516) in a separate group, and excluded N = 100 participants who picked the category ‘Other’ (e.g., spiritual, pagan, Wiccan, etc.). A 2 religion (some religion, Atheist/Agnostic) X 2 participant sex (Female, Male) ANOVA with GCB scores as response variable showed main effects of religion (*F*(1,1207) = 77.699, *p* < 0.0001, *η*^2^_g_ = 0.060), and participant sex (*F*(1,1207) = 50.798, *p* < 0.0001, *η*^2^_g_ = 0.040), but no interaction, (*p* > 0.6). See Fig 3D. Post hoc tests showed that while in both religious and non-religious groups, females had higher GCB than males, this difference was smaller in religious participants (some religion: *M*_*diff*_ = 11.41 *SE* = 2.72, *t*(1207) = 4.192, *p* = 0.0002, Atheist/Agnostic: *M*_*diff*_ = 19.24, *SE* = 3.17, *t*(1207) = 6.061, *p* < .0001). Interestingly, the Atheist/Agnostic males did not significantly differ from religious female participants in GCB (*M*_*diff*_ = 3.24 *SE* = 2.77, *t*(1207) = 1.170, *p* = 1). See Section D in S1 File for details and pairwise comparison plots.

Finally, we looked at geographical regions of residence. We categorized the states where the subject resided into 4 groups (see Section A in S1 File) and tested an ANOVA model predicting GCB with 2 participant sex (Female, Male) X 4 region (*Northeast*, *Midwest*, *South*, and *West*.). Results showed significant main effects of participant sex and region but no interaction (See Section D in S1 File). Post hoc tests with Bonferroni correction showed that the gender difference in GCB was robust in every region except for the Midwest (*Northeast*: *M*_*diff*_ = 14.33, 95%CI[4.53, 28.22], *p* = 0.0372), *South*: (*M*_*diff*_ = 12.78, 95%CI[23.02, 25.36], *p* = 0.0039), *West*: (*M*_*diff*_ = 17.25, 95%CI[4.20, 30.31], *p* = 0.0016), *Midwest*: (*M*_*diff*_ = 7.41, 95%CI[5.83, 20.64], *p* = 0.6872). See Fig 3E.

In summary, gender category beliefs are influenced by age, participant sex, political ideology, religion, and region of residence, which highlights the importance of social circumstance and lived experience in the formation of gender category beliefs. With respect to age, it is unclear whether the decrease in GCB is associated with age or the participants’ cohort. Liberal women tend to believe that gender is more self-described than liberal men, but conservative men and women have indistinguishable gender category beliefs. This suggests that in addition to lived experiences and motives to maintain the boundaries between genders, political ideology shapes beliefs about the nature of gender groups. However, it is unclear to what extent this is signaling group affiliation, and to what extent is it a product of a general belief system and its prescribed social order.

In both studies, participants gave their views on transgender men and transgender women’s use of bathrooms that match their self-identified genders, which prior work shows are related to warm-cold feelings towards transgender people [5]. Using bathroom of choice was considered wrong by 29.2% of participants and not wrong by 70.7%. Male participants were slightly more likely to view transgender people's use of the bathroom that matches their identified gender as wrong (Study 1: F/M = 24.6%/31.7%; Study 2: F/M = 27.6%/33.4%) but these differences were not significant (*p* > 0.05).

Policy preferences were re-coded (wrong/not wrong: 0/1) and regressed on GCB and affect scores using logistic regression models. Results showed that in both Study 1 and Study 2, GCB predicted policy preferences (McFadden *R*^*2*^s: Study 1 = 0.259; Study 2 = 0.366) and so did thermometer ratings (McFadden *R*^*2*^s: Study 1 = 0.180; Study 2 = 0.332). In the model that included both scores, GCB and warmth were both significant predictors of views toward bathroom choice, (Study 1: McFadden *R*^*2*^s = 0.309, *b*_*GCB*_ = 0.031, *SE* = 0.003; *b*_*warmth*_ = 0.024, *SE* = 0.004; Study 2: McFadden *R*^*2*^s = 0.464, *b*_*GCB*_ = 0.037, *SE* = 0.004; *b*_*warmth*_ = 0.041, *SE* = 0.005, *P*s < 0.0001). In the pooled sample, controlling for study, for each unit increase in GCB, the odds of approving (versus disapproving) transgender people’s use bathroom that matches their identified gender increases by a factor of 1.033 (95%CI[1.03, 1.041]). Same odds increase by a factor of 1.035 (95%CI[1.02, 1.04]) for every increase in the warmth scores. There was no effect of study (*p* < 0.3). See Section F in S1 File for details of these results.

We further examined this comparison using 10-fold cross-validation on the pooled sample. In sum, we split the dataset into 10 random segments (i.e. folds), and extracted estimates using logistic regression models from 9 of those segments. We then used these estimates to predict responses in the remaining one segment, and compared the predictions with the participants' true bathroom choice preferences (i.e., we examined each model’s ability to predict 0s as 0s and 1s as 1s). We did this 10 times (i.e., 10 folds) and for three different models: GCB, Warmth, GCB+Warmth. The model with GCB as a predictor outperformed the model with Warmth as a predictor (Mean AUC: 89.2% vs. 81.8%) in almost every fold. See Fig 3B. That is, GCB scores were better than feelings at distinguishing between people who were for or against bathroom use. Out of all combinations of GCB, its constituents and Warmth, the worst performance belonged to the model with warmth scores alone, and the best performance was obtained from the model that included both GCB and warmth (See Section F in S1 File for detailed results and comparison of different models).

These results suggest that while GCB and affect towards transgender people overlap, they also contribute separately to policy preferences. GCB scores capture unique variance in policy preferences. As outlined in the introduction, this might be because people disagree with the right to bathroom choice but underreport negative attitudes, or because negative attitudes do not always mean approval of discriminatory policies.

## Discussion

Anti-transgender sentiments, our results imply, are strongly associated with the view that gender is determined at birth and is based on genes or genitals or other observable characteristics [62]. When coming from an institution and adopted as a policy, this view could also shift the perceptions of social norms, which are powerful drivers of behavior [63]. It could adversely impact the lives of transgender people who already face increased risk of discrimination in education and hiring, higher poverty rates, and lack of access to psychological and medical services on which they rely [4,64], and have alarmingly high rates of suicide and likelihood of experiencing sexual assault [65,66].

Our results show that folk theories of gender and affect toward transgender people each capture unique variance in transgender bathroom policy preferences. We tested three models with different predictors: affect, gender category beliefs, and affect plus gender category beliefs. For each model, we used the estimates from one segment of the sample to predict responses in another segment of the sample. Comparison of predicted preferences with observed preferences shows that the model with both gender category beliefs and affect accurately predicted bathroom policy preferences in almost 90% of cases. Gender category beliefs and affect were each accurate about 85% and 80% of cases respectively.

A possible reason for this improvement in predicting policy positions is the complex relationship between affect, beliefs and policy preferences. Affect and beliefs might be related to different forms of discrimination [36], or depending on contextual and demographic factors, policy preferences could more belief-driven or more affect-driven [37]. Another explanation might be that people do not truthfully report their feelings about transgender people, or they may not harbor negative feelings toward them but still oppose bathroom choice right as a policy. While self-representation bias could prevent people from stating their true feelings, beliefs about gender offer a seemingly less prejudicial means to justify and maintain their policy positions. In other words, beliefs might have can be measured with less error. Alternatively, a person might not particularly like or dislike targets of discriminatory policies yet conform to the policy positions endorsed by their ideological, religious or other identity groups. In either case, these results suggest that beliefs about gender capture variance in policy positions unexplained by affect alone.

Female perceivers are more willing than males to categorize transgender people by the self-identified gender. This is independent of the type or the direction of the gender transition procedure, which suggests that beliefs about gender are not mere outcomes of evolutionary or cognitive imperatives [24]. They are, at least in part, the products of one’s experience and position within social structures, and their motives to defend or oppose the social order. This is corroborated by our findings that theories of gender vary widely based on geography, political ideology, religiosity, and age. The gender difference is consistent with theoretical work on motivated perception and attribution [53,67–69], and research on the relationship between attitudes toward gender egalitarianism and social dominance orientation ([53,67–69]). High-status members of a given hierarchy strive to maintain the hierarchy.

Biology seems to carry a significant weight in how people determine someone’s gender, with more favorable attitudes toward transgender individuals who undergo medical procedures to align their biology with their gender identity. This might be due to reduced ambiguity, ease of categorization [20], and/or reduced perceptions of threat to the gender binary. It could also be that when people imagine an invasive hormonal and surgical procedure, with this honest signal of effort and expense, they realize that a transgender person is not simply choosing their gender but correcting an essential characteristic that was misidentified early on.

We also find that gender category beliefs, or theories that underlie people’s judgments of another person’s gender, are extremely polarized. About half of our sample view gender as either fully determined at birth and fixed, or completely determined by the self. Concern for transgender affairs and rights seems to signal ideological affiliation. Attention to transgender issues and rights in political discourse has been growing in recent years, which has led to increased polarization in transgender policy stances between the political left and right [70]. Our analysis confirms this by showing a consistent decrease in self-described views of gender towards the right of the political ideology spectrum and its demographic correlates (e.g., age, religiosity). Moreover, gender differences in the beliefs that gender is self-described decrease as conservatism increases. Future work could investigate to what degree this is motivated and a signal of group affiliation, or it is a learned descriptive or prescriptive belief.

These findings have practical implications for interventions that target support for anti-transgender policies. Approaches that focus on feelings such as evoking empathy can be accompanied with approaches that target beliefs to reach a larger audience with diverse demographics. The more educated participants, for example, might be more predisposed to belief-focused interventions. Moreover, the significant role of biology in folk theories of gender might seemingly suggest that if people think about biological transition processes, they might adopt a more positive attitude toward transgender people. Nonetheless, this type of intervention could endanger the psychological well-being and safety of transgender individuals who are pre-operative, do not wish to undergo medical interventions, or lack access to medical interventions to align their gender identity with their biology. It burdens the individual with the notion that if they really mean it, then they should be willing to undergo surgery, and may inadvertently focus hostility towards those who do not.

Overall, lay theories act as guides to interpret social worlds. They powerfully direct interpersonal behavior [71], and are consequential for group perception and preferences for intergroup engagement [58,72]. Experiments have shown that targeting lay beliefs can shift people’s views of the groups involved in the Palestinian-Israeli conflict, reduce negative attitudes, and increase support to compromise for peace [42]. Relatedly, implicit theories about the malleability of individuals [73] and social groups [74] influence how they are perceived and stereotyped in consequential ways. Beliefs about the nature of groups are closely tied to how we choose to engage with them and attitudes about how they should be treated. More rigid beliefs about boundaries are associated with discriminatory policies [75].

A limitation of our research is that we did not measure the sexual orientation of our participants, nor do we have data on their contact with transgender people [76,77]. Knowledge about gender and education could also shift a person’s belief about gender and/or attitudes toward transgender people and sexual minorities [78]. In a similar vein, given that estimates of the transgender population are less than 1% [79], one can assume that the vast majority of participants are cisgender, even though we measure participant gender using a tri-category scale (Female/Male/Other). Future studies should include response options that allow participants to indicate whether they are transgender or cisgender. Future work could also shed light on folk theories of gender by focusing on samples that vary along these dimensions. More importantly, sampling from non-WEIRD populations could provide valuable insight into the universal and cultural factors that shape conceptions of gender [80,81].

Future studies could also expand the outcome measures being predicted by affect and gender category beliefs beyond policy beliefs about bathroom access. This issue has been salient due to publicity around the Supreme Court case of Gavin Grimm, a transgender high school student who sued his school for preventing him from using the boys’ bathroom [82], as well as individual states that attempted to (some successfully) pass legislation restricting transgender bathroom access [83]. However, there are myriad other ways in which the transgender community faces discrimination in public and private life, and should be included as outcome measures in future studies. These could include issues regarding officialized gender on identification documents (e.g., passport, driver's license) or at institutions (prisons, hospitals). Other important LGBT policy issues that could be included in future studies are currently being decided by the U.S. Supreme Court [84–86]. These could include whether it is legally permissible or to how acceptable is it to fire a transgender person from their job for being transgender, deny a transgender person housing, refuse to serve a transgender person in public accommodations (e.g. restaurants, hotels), and refuse to treat a transgender person in a health care setting citing violations of one’s deeply held religious beliefs.

## Supporting information

S1 File Includes (Section A in S1 File) sample characteristics, (Section B in S1 File) study material, exclusions, and power analysis, (Section C in S1 File) analysis of the folk theories of gender tasks, (Section D in S1 File) analysis of demographic variables, (Section E in S1 File) analyses of feeling thermometer tasks, (Section F in S1 File) analysis of the bathroom policy question, (Section G in S1 File) complementary analyses, and (Section H in S1 File) content analysis of the open-ended responses.
(DOCX)
