# Supplementary material for: Folk theories of gender and anti-transgender attitudes: Gender differences and policy preferences
Source: PLoS One. 2019 Dec 30;14(12):e0226967. doi: 10.1371/journal.pone.0226967 (PMC6936834; doi:10.1371/journal.pone.0226967)
Supplement: S1 File — Includes (Section A in S1 File) sample characteristics, (Section B in S1 File) study material, exclusions, and power analysis, (Section C in S1 File) analysis of the folk theories of gender tasks, (Section D in S1 File) analysis of demographic variables, (Section E in S1 File) analyses of feeling thermometer tasks, (Section F in S1 File) analysis of the bathroom policy question, (Section G in S1 File) complementary analyses, and (Section H in S1 File) content analysis of the open-ended responses. (DOCX) [file pone.0226967.s001.docx]

Supporting Information for

Folk Theories of Gender and Anti-Transgender Attitudes: Gender Differences and Policy Preferences

Mostafa Salari Rad ^1,2,3, *^

Crystal Shackleford ^3^

Kelli Ann Lee ^3^

Kate Jassin ^3^

Jeremy Ginges ^3^

^1^ Kahneman-Treisman Center for Behavioral Science & Public Policy, Woodrow Wilson School of Public and International Affairs, Princeton University, Princeton, NJ, United States of America
^2^ Department of Psychology, Princeton University, Princeton, NJ, United States of America

^3^ Department of Psychology, New School for Social Research, The New School, New York City, United States of America

* Corresponding author: Mostafa Salari Rad,

E-mail: salam9955@newschool.edu

[A. Samples 3](#_Toc532912052)

[1.1 Study 1 3](#_Toc532912053)

[1.2 Study 2 4](#_Toc532912054)

[B. Instrument and design 5](#_Toc532912055)

[2.1 Study 1 5](#_Toc532912056)

[2.2 Study 2 6](#_Toc532912057)

[2.3 Exclusions 7](#_Toc532912058)

[2.4 Power analysis 7](#_Toc532912059)

[C. Detailed Results 8](#_Toc532912060)

[3.1 Study 1 8](#_Toc532912061)

[3.2 Study 1 - Gender Category Beliefs (GCB) 9](#_Toc532912062)

[3.3 Study 2 10](#_Toc532912063)

[3.4 Study 2 - Gender Category Beliefs (GCB) 11](#_Toc532912064)

[3.3 Results of Study 1 & 2 (Plot) 12](#_Toc532912065)

[D. Demographic Breakdown 13](#_Toc532912066)

[4.1 Age 13](#_Toc532912067)

[4.2 Political Ideology 15](#_Toc532912068)

[4.3 Religion 17](#_Toc532912069)

[4.4 Race 18](#_Toc532912070)

[4.5 Geographical Region 19](#_Toc532912071)

[4.6 Combined Demographics 20](#_Toc532912072)

[E. Feelings toward Transgender People 22](#_Toc532912073)

[5.1 Feelings and GCB 23](#_Toc532912074)

[F. Bathroom Choice Responses 25](#_Toc532912075)

[6.1 Feelings vs. GCB – Logistic Regressions 25](#_Toc532912076)

[6.2 Feelings vs. GCB – Cross Validation 27](#_Toc532912077)

[G. Additional Analyses 28](#_Toc532912078)

[H. Open-ended Responses 30](#_Toc532912079)

## A. Samples

### 1.1 Study 1

| Sample | N = 623 | | | | | | |
| --- | --- | --- | --- | --- | --- | --- | --- |
| Sex (N) | F:329/M:294 | | | | | | |
| Age (%) | 18-25 | 26-34 | | 35-54 | | 55-64 | 65+ |
|  | 20.5 | 37.4 | | 31.5 | | 7.9 | 2.7 |
| State (%) | Mid-west | Northeast | | South | | West | |
|  | 19 | 19.6 | | 37.4 | | 24 | |
| Political Ideology (%) | (1)  very liberal | (2) | (3) | (4) | (5) | (6) | (7)  very conservative |
|  | 15.9 | 19.1 | 15.4 | 24.4 | 11.1 | 7.9 | 6.3 |
| Religion (%) | Some Religion: 54.6 | | Christian: 48  Muslim: 1.1  Jewish: 2.1  Hindu: 1.3  Buddhist: 1.4  Mormon: 0.6 | | | | |
|  | Atheist/Agnostic: 36 | |  |  |  |  |  |
|  | Other: 9.5 | |  |  |  |  |  |
| Race (%) | White | Black | Hispanic | Asian | Native American | Pacific Islander | Other |
|  | 76.4 | 7.1 | 6.6 | 6.9 | 1.3 | 0.2 | 1.6 |

**Midwest**: Illinois, Indiana, Iowa, Kansas, Michigan, Minnesota, Missouri, North South Dakota, Nebraska, Ohio, Wisconsin

**South:** Alabama, Florida, Georgia, Kentucky, Mississippi, North Carolina, South Carolina, Tennessee, Maryland, Virginia, West Virginia, Oklahoma

**Northeast**: Maine, New York, New Jersey, Vermont, Massachusetts, Rhode Island, Connecticut, New Hampshire, Pennsylvania

**West**: Alaska, Arizona, California, Colorado, Hawaii, Idaho, Montana, Nevada, New Mexico, Oregon, Utah, Washington, Wyoming

### 1.2 Study 2

| Sample | N = 702 | | | | | | |
| --- | --- | --- | --- | --- | --- | --- | --- |
| Sex (N) | F:352/M:350 | | | | | | |
| Age (%) | 18-25 | 26-34 | | 35-54 | | 55-64 | 65+ |
|  | 14.9 | 39.3 | | 33.6 | | 8.9 | 3.4 |
| State (%) | Mid-west | Northeast | | South | | West | |
|  | 24 | 19.7 | | 35.6 | | 20.7 | |
| Political Ideology (%) | (1)  very liberal | (2) | (3) | (4) | (5) | (6) | (7)  very conservative |
|  | 17.3 | 20.0 | 14.1 | 20.6 | 12.7 | 10.4 | 4.9 |
| Religion (%) | Some Religion: 50.7 | | Christian: 42.6  Muslim: 0.9  Jewish: 1.7  Hindu: 2.3  Buddhist: 2.4  Mormon: 0.9 | | | | |
|  | Atheist/Agnostic: 41.7 | |  |  |  |  |  |
|  | Other: 7.6 | |  |  |  |  |  |
| Race (%) | White | Black | Hispanic | Asian | Native American | Pacific Islander | Other |
|  | 74.7 | 6.7 | 5.7 | 10.0 | 1.1 | 0 | 1.7 |

####

## B. Instrument and design

####

### 2.1 Study 1

| Factorial design: Frame X Label X Transformation X Direction | | |
| --- | --- | --- |
| Type | Factor | Levels |
| Between subject | Transformation | Non-biological:  *Jack was born with the sexual characteristics of a male and was raised as a boy. As Jack grew up, Jack began to identify more and more as a woman. When Jack became an adult, Jack stopped going by their legal name, and started answering to the name of “Jill”. Jill grew long hair, likes to wear clothes that are thought of as feminine, and wears lipstick to work.* |
|  |  | Biological:  *‘Jill was born with the sexual characteristics of a female and was raised as a girl. As Jill grew up, Jill began to identify more and more as a man. When Jill became an adult, Jill stopped going by their legal name, and started answering to the name of “Jack”. Jack’s hair is now cut very short, and Jack likes to wear clothes that are thought of as masculine. After much thought and counselling, Jack decided to undergo hormone therapy, surgical removal of breasts, and surgical construction of a penis.’* |
|  | Direction | Male to female, Female to male (reversed names above) |
| Within subject | Frame ^1^ | Assigned gender, Identified gender |
|  | Label | Male/Female, Man/Woman |
| *To what extent do* ***you*** *think Jack/Jill is a Man, Woman, Male, Female? ^2^*  *(0% - 10% - 20% - 30% - 40% - 50% - 60% - 70% - 80% -90% - 100%)*  *Can you explain your reasoning?* | | |

^1^ Frame is based on the condition. For example, in the male-to-female scenario, when we ask participants about the extent to which they think that Jack/Jill is a man, we are asking about the degree to which the person has the assigned-at-birth gender. All things equal, but in the reversed direction of transformation, we would be asking about the degree to which the person has the self-identified gender. By using this wording we do not mean gender has necessarily changed.

^2^ Four separate sets of questions (rating & open-ended probe) were asked on four different pages.

### 2.2 Study 2

| Factorial design: Frame X Label X Transformation X Direction | | |
| --- | --- | --- |
| Type | Factor | Levels |
| Between subject | Transformation | Non-biological:  *Patricia was born with the sexual characteristics of a female and was raised as a girl. As Patricia grew up, Patricia began to identify more and more as a man. When Patricia became an adult, Patricia stopped going by their legal name, and started answering to the name of "Patrick". Patrick's hair is now cut very short, and Patrick likes to wear clothes that are thought of as masculine.* |
|  |  | Biological:  *Patricia was born with the sexual characteristics of a female and was raised as a girl. As Patricia grew up, Patricia began to identify more and more as a man. When Patricia became and adult, Patricia stopped going by their legal name, and started answering to the name of "Patrick". Patrick's hair is now cut very short, and Patrick likes to wear clothes that are thought of as masculine. After much thought and counseling, Patrick decided to undergo hormone therapy, surgical removal of breasts, and surgical construction of a penis.* |
|  | Direction | Male to female, Female to male (reversing names above) |
| Within subject | Frame | Assigned gender, Identified gender |
|  | Label | Male/Female, Man/Woman |
| *To what extent do* ***you*** *think this individual is a Man, Woman, Male, Female?*  *(0% - 10% - 20% - 30% - 40% - 50% - 60% - 70% - 80% -90% - 100%)*  *Can you explain your reasoning?* ^3^ | | |

^3^ We added a number of scales for exploratory purposes at the end of this survey. These scales are listed in the preregistration on the OSF website ([here](https://osf.io/m7vhk/)) but we will not report or analyze them in this paper.

### 2.3 Exclusions

We included multiple attention check items in the survey and survey completion codes. Some participants answered incorrectly to these items, or missed survey completion codes. Also, we asked participants to explain their answers in a few sentences. This open-ended probe provided another means of detecting inattentive responses, lack of comprehension, and responses generated by bots. Reported results are based on the fully attentive subsamples who indicated their nationality as American and their open-ended responses were not random.

We also excluded participants that did not identify as male or female (Study 1: 4, Study 2: 8), participants who were not from the United States, or their location was not identifiable (Study 1: 12, Study 2: 5), or did not correctly submit the randomly generated completion coed provided at the end of the study (Study 1: 12, Study 2: 5). Using Turkprime, we ensured participants from Study 1 were not able to participate in Study 2.

### 2.4 Power analysis

Our experiment was 2 X 2 (Direction and Transformation) between-subject design, not considering participant gender. We measured 4 variables within subject (Frame, Label). Focusing on the interaction between Frame and other factors, power analysis indicated that in order to detect an interaction of size of $\eta$^2^_partial_ = 0.02 with 0.9 power, we would need a sample size of 656. In Study 1, exclusions resulted in smaller sample (N = 623). In Study 2, we over recruited and our sample became larger (N = 700). See preregistration on the OSF website ([here](https://osf.io/m7vhk/)).

## C. Detailed Results

###

### 3.1 Study 1

*gender rating ~ transformation * scenario (i.e. direction) * participant sex * frame * label*

Using ezANOVA:

dv = .(value),

wid = .(ResponseID),

within = .(label, frame),

between = .(transformation, scenario, participant.sex)

| **Effect** | **DFn** | **DFd** | **F** | **p** | **ges^1^** | **p<.05** |
| --- | --- | --- | --- | --- | --- | --- |
| transformation | 1 | 619 | 0.7955 | 0.3728 | 0.0001 |  |
| scenario | 1 | 619 | 0.0244 | 0.8759 | 0 |  |
| participant.sex | 1 | 619 | 0.0002 | 0.9881 | 0 |  |
| label | 1 | 619 | 3.5151 | 0.0613 | 0.0002 |  |
| frame | 1 | 619 | 1.7552 | 0.1857 | 0.0024 |  |
| transformation:scenario | 1 | 619 | 0.0795 | 0.7781 | 0 |  |
| transformation:participant.sex | 1 | 619 | 0.2111 | 0.6461 | 0 |  |
| scenario:participant.sex | 1 | 619 | 0.0077 | 0.9301 | 0 |  |
| transformation:label | 1 | 619 | 0.1161 | 0.7334 | 0 |  |
| scenario:label | 1 | 619 | 0 | 0.9955 | 0 |  |
| participant.sex:label | 1 | 619 | 0.6583 | 0.4175 | 0 |  |
| **transformation:frame** | **1** | **619** | **19.5806** | **0** | **0.0266** | ***** |
| scenario:frame | 1 | 619 | 0.8314 | 0.3622 | 0.0012 |  |
| **participant.sex:frame** | **1** | **619** | **32.2029** | **0** | **0.0431** | ***** |
| **label:frame** | **1** | **619** | **9.3461** | **0.0023** | **0.0009** | ***** |
| transformation:scenario:participant.sex | 1 | 619 | 0.4836 | 0.4871 | 0 |  |
| transformation:scenario:label | 1 | 619 | 1.4185 | 0.2341 | 0.0001 |  |
| transformation:participant.sex:label | 1 | 619 | 1.6252 | 0.2028 | 0.0001 |  |
| scenario:participant.sex:label | 1 | 619 | 0.0616 | 0.8041 | 0 |  |
| transformation:scenario:frame | 1 | 619 | 0.0584 | 0.8091 | 0.0001 |  |
| transformation:participant.sex:frame | 1 | 619 | 0.0214 | 0.8836 | 0 |  |
| scenario:participant.sex:frame | 1 | 619 | 0.0697 | 0.7919 | 0.0001 |  |
| transformation:label:frame | 1 | 619 | 0.1594 | 0.6899 | 0 |  |
| scenario:label:frame | 1 | 619 | 0.3942 | 0.5303 | 0 |  |
| participant.sex:label:frame | 1 | 619 | 0.0957 | 0.7571 | 0 |  |
| transformation:scenario:participant.sex:label | 1 | 619 | 1.2828 | 0.2578 | 0.0001 |  |
| transformation:scenario:participant.sex:frame | 1 | 619 | 0.0884 | 0.7663 | 0.0001 |  |
| transformation:scenario:label:frame | 1 | 619 | 2.6136 | 0.1065 | 0.0002 |  |
| transformation:participant.sex:label:frame | 1 | 619 | 0.05 | 0.8231 | 0 |  |
| scenario:participant.sex:label:frame | 1 | 619 | 0.5007 | 0.4794 | 0 |  |
| transformation:scenario:participant.sex:label:frame | 1 | 619 | 0.1251 | 0.7236 | 0 |  |

^1^ Generalized Eta squared

### 3.2 Study 1 - Gender Category Beliefs (GCB)

| raw_alpha | std.alpha* | mean | sd |
| --- | --- | --- | --- |
| 0.95 | 0.95 | 5.3 | 3.7 |

* Same for male and female participants.

*gender rating ~ transformation * scenario (i.e. direction) * participant sex*

| **Effect** | **DFn** | **DFd** | **F** | **p** | **ges** | **p<.05** |
| --- | --- | --- | --- | --- | --- | --- |
| **transformation** | **1** | **615** | **19.6114** | **0** | **0.0309** | ***** |
| scenario | 1 | 615 | 0.5984 | 0.4394 | 0.00097 |  |
| **participant.sex** | **1** | **615** | **32.8311** | **0** | **0.050** | ***** |
| transformation:scenario | 1 | 615 | 0.0414 | 0.8387 | 0.00006 |  |
| transformation:participant.sex | 1 | 615 | 0.0156 | 0.9003 | 0.00002 |  |
| scenario:participant.sex | 1 | 615 | 0.0163 | 0.8984 | 0.00002 |  |
| transformation:scenario:participant.sex | 1 | 615 | 0.0600 | 0.8064 | 0.00009 |  |

### 3.3 Study 2

| **Effect** | **DFn** | **DFd** | **F** | **p** | **ges** | **p<.05** |
| --- | --- | --- | --- | --- | --- | --- |
| transformation | 1 | 694 | 0.0118 | 0.9135 | 0 |  |
| scenario | 1 | 694 | 0.0001 | 0.9904 | 0 |  |
| participant.sex | 1 | 694 | 0.2222 | 0.6375 | 0 |  |
| label | 1 | 694 | 0.0381 | 0.8452 | 0 |  |
| frame | 1 | 694 | 7.359 | 0.0068 | 0.0093 |  |
| transformation:scenario | 1 | 694 | 0.0051 | 0.943 | 0 |  |
| transformation:participant.sex | 1 | 694 | 0.1504 | 0.6983 | 0 |  |
| scenario:participant.sex | 1 | 694 | 0.0254 | 0.8734 | 0 |  |
| transformation:label | 1 | 694 | 1.0738 | 0.3004 | 0 |  |
| scenario:label | 1 | 694 | 1.5873 | 0.2081 | 0 |  |
| participant.sex:label | 1 | 694 | 3.6867 | 0.0553 | 0.0001 |  |
| **transformation:frame** | **1** | **694** | **20.1384** | **0** | **0.025** | ***** |
| scenario:frame | 1 | 694 | 0.0466 | 0.8291 | 0.0001 |  |
| **participant.sex:frame** | **1** | **694** | **11.2961** | **0.0008** | **0.0142** | ***** |
| **label:frame** | **1** | **694** | **14.3719** | **0.0002** | **0.0009** | ***** |
| transformation:scenario:participant.sex | 1 | 694 | 0.0381 | 0.8453 | 0 |  |
| transformation:scenario:label | 1 | 694 | 2.6265 | 0.1055 | 0.0001 |  |
| transformation:participant.sex:label | 1 | 694 | 0.24 | 0.6244 | 0 |  |
| scenario:participant.sex:label | 1 | 694 | 1.2504 | 0.2639 | 0 |  |
| transformation:scenario:frame | 1 | 694 | 2.0128 | 0.1564 | 0.0026 |  |
| transformation:participant.sex:frame | 1 | 694 | 0.3051 | 0.5809 | 0.0004 |  |
| scenario:participant.sex:frame | 1 | 694 | 2.8286 | 0.0931 | 0.0036 |  |
| transformation:label:frame | 1 | 694 | 0.9163 | 0.3388 | 0.0001 |  |
| scenario:label:frame | 1 | 694 | 0.035 | 0.8517 | 0 |  |
| participant.sex:label:frame | 1 | 694 | 0.0655 | 0.7981 | 0 |  |
| transformation:scenario:participant.sex:label | 1 | 694 | 0.0374 | 0.8468 | 0 |  |
| transformation:scenario:participant.sex:frame | 1 | 694 | 1.2811 | 0.2581 | 0.0016 |  |
| transformation:scenario:label:frame | 1 | 694 | 0.0085 | 0.9266 | 0 |  |
| transformation:participant.sex:label:frame | 1 | 694 | 0.0832 | 0.7731 | 0 |  |
| scenario:participant.sex:label:frame | 1 | 694 | 0.7785 | 0.3779 | 0.0001 |  |
| transformation:scenario:participant.sex:label:frame | 1 | 694 | 0.5018 | 0.479 | 0 |  |

####

###

### 3.4 Study 2 - Gender Category Beliefs (GCB)

| raw_alpha | std.alpha | mean | sd |
| --- | --- | --- | --- |
| 0.95 | 0.95 | 5.4 | 3.7 |

* Same for male and female participants.

*gender rating ~ transformation * scenario (i.e. direction) * participant sex*

| **Effect** | **DFn** | **DFd** | **F** | **p** | **ges** | **p<.05** |
| --- | --- | --- | --- | --- | --- | --- |
| **transformation** | **1** | **692** | **19.5935** | **0** | **0.0275** | ***** |
| scenario | 1 | 692 | 0.0197 | 0.8882 | 0 |  |
| **participant.sex** | **1** | **692** | **11.4479** | **0.0007** | **0.0162** | ***** |
| transformation:scenario | 1 | 692 | 2.0514 | 0.1525 | 0.0029 |  |
| transformation:participant.sex | 1 | 692 | 0.3197 | 0.5719 | 0.0004 |  |
| scenario:participant.sex | 1 | 692 | 2.7400 | 0.0983 | 0.0039 |  |
| transformation:scenario:participant.sex | 1 | 692 | 1.2847 | 0.2574 | 0.0018 |  |

###

### 3.3 Results of Study 1 & 2 (Plot)

Figure 1. Y-axis he character’s gender, split by Frame (Assigned gender, Identified gender), Study, Transition Type (biological vs. non-biological), Transition Direction (Assigned M-Identified-F vs. Assigned-F-Identified-M), and Label (man/woman vs. male/female).


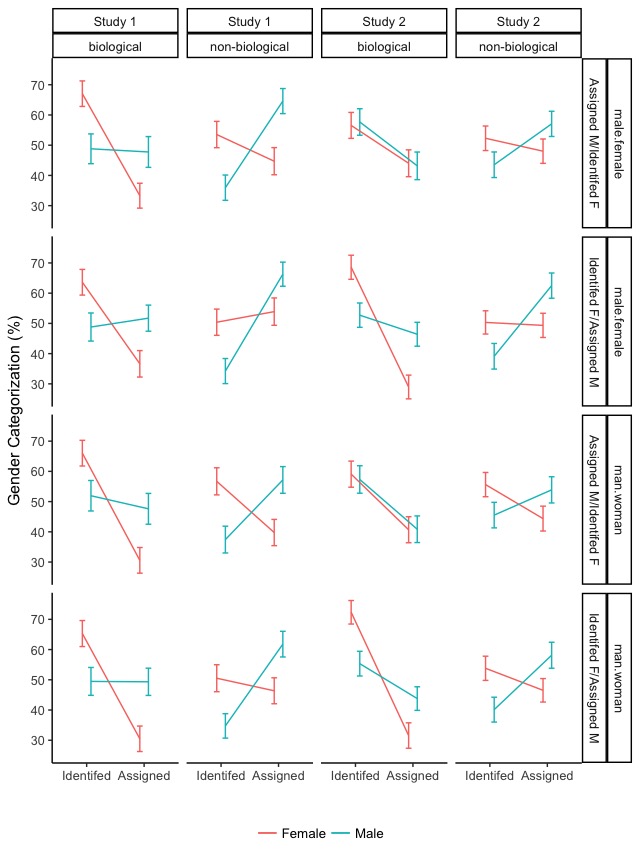


## D. Demographic Breakdown

In addition to participant gender, we examine variance in GCB as function of age, political ideology, religion, race, and geographical region, and a combination of these variables. We report this analysis for the combined sample from the two studies to increase statistical power. For each variable, we provide descriptive statistics, test its association with GCB in models where participant gender is also a predictor, and plot the pairwise comparisons.

### 4.1 Age

We combined age groups 55-65 and 65< since there were too few observations. This variable was distributed as follows: 18-25 (17.54%), 26-34 (38.40%), 35-55 (32.58%), 55< (11.49 %).

*GCB ~ participant gender * age group*

| **Effect** | **DFn** | **DFd** | **F** | **p** | **ges** | **p<.05** |
| --- | --- | --- | --- | --- | --- | --- |
| **age** | 3 | 1315 | 6.1521 | 3.75E-04 | 0.01384102 | * |
| **participant.sex** | 1 | 1315 | 45.940 | 1.83E-11 | 0.03375677 | * |
| participant.sex:age | 3 | 1315 | 1.0135 | 3.86E-01 | 0.00230702 |  |

We find no significant interaction between participant gender and age (Figure 2). Treating age as a continuous variable, we find the association between gender and GCB to be stronger in females (Spearman $\rho$= -0.15, *P* < 0.0001) than males (Spearman $\rho$= -0.09, *P* = 0.01).

Figure 2. GCB by age and participant gender. Numbers on median lines are cell sizes.
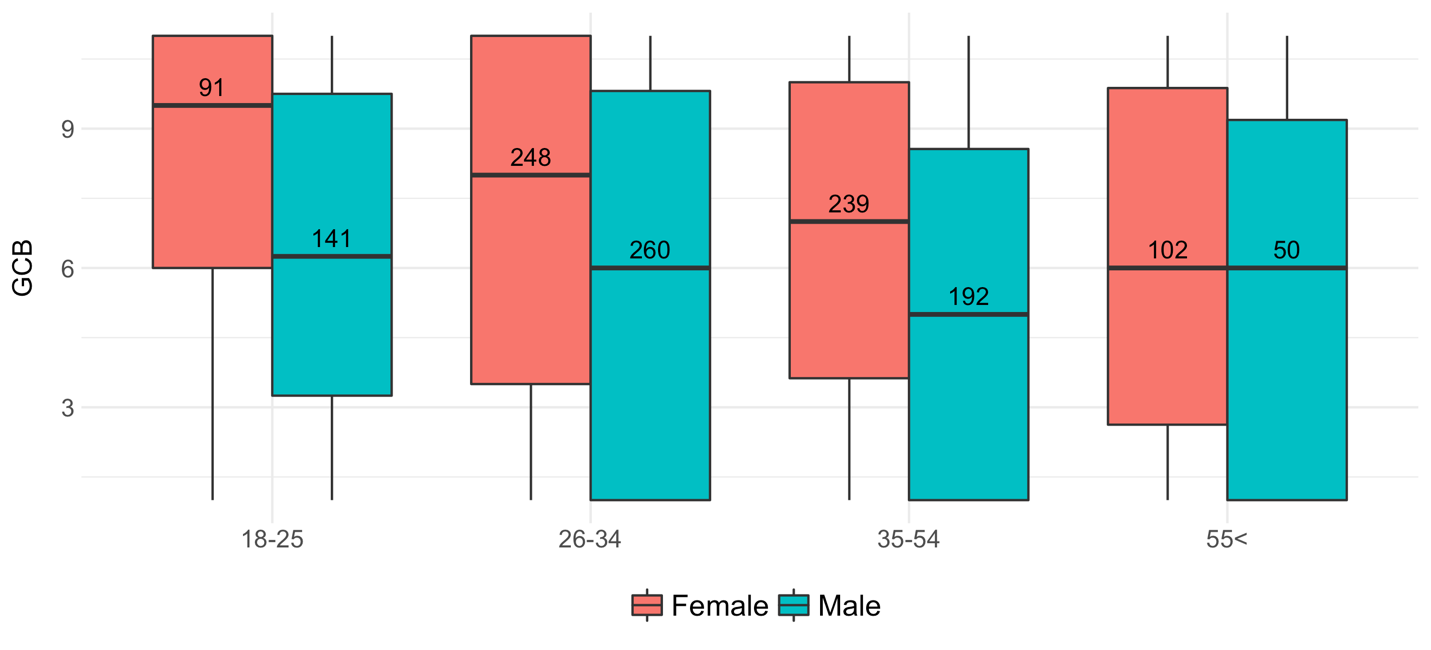


Figure 3. Post hoc pairwise comparison with Bonferroni correction (*Participant sex * Age*).


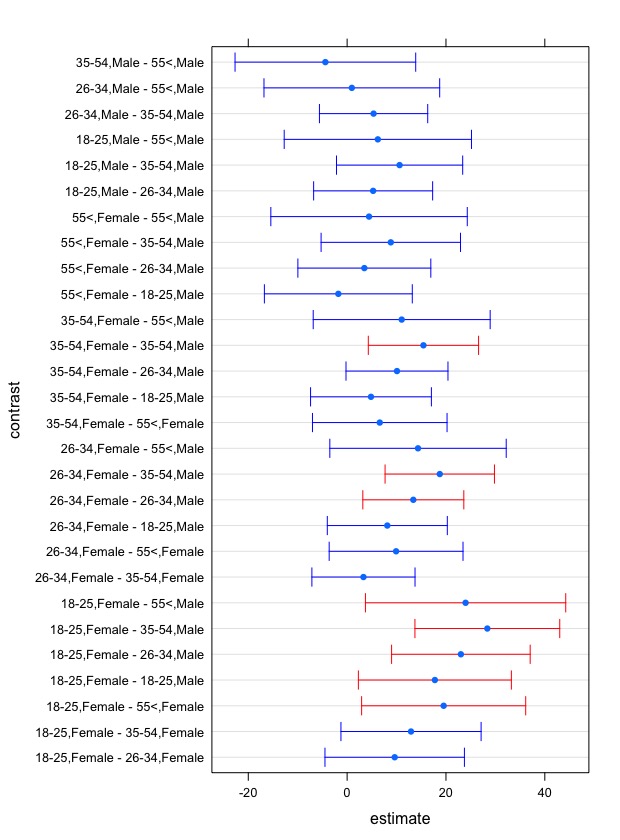


### 4.2 Political Ideology

We measured political ideology on a 7-point scale (*very liberal* 1 - 7 *very conservative*).

*GCB ~ participant gender * political ideology*

| **Effect** | Df | Sum Sq | Mean Sq | F value | Pr(>F) |  |
| --- | --- | --- | --- | --- | --- | --- |
| **political.ideology** | **1** | **421951** | **421951** | **395.1606** | **2.20E-16** | ***** |
| **participant.sex** | **1** | **28875** | **28875** | **27.0412** | **2.31E-07** | ***** |
| political.ideology:participant.sex | 1 | 492 | 492 | 0.4604 | 0.4975 |  |

Figure 4. GCB as a function of political ideology, split by participant gender.


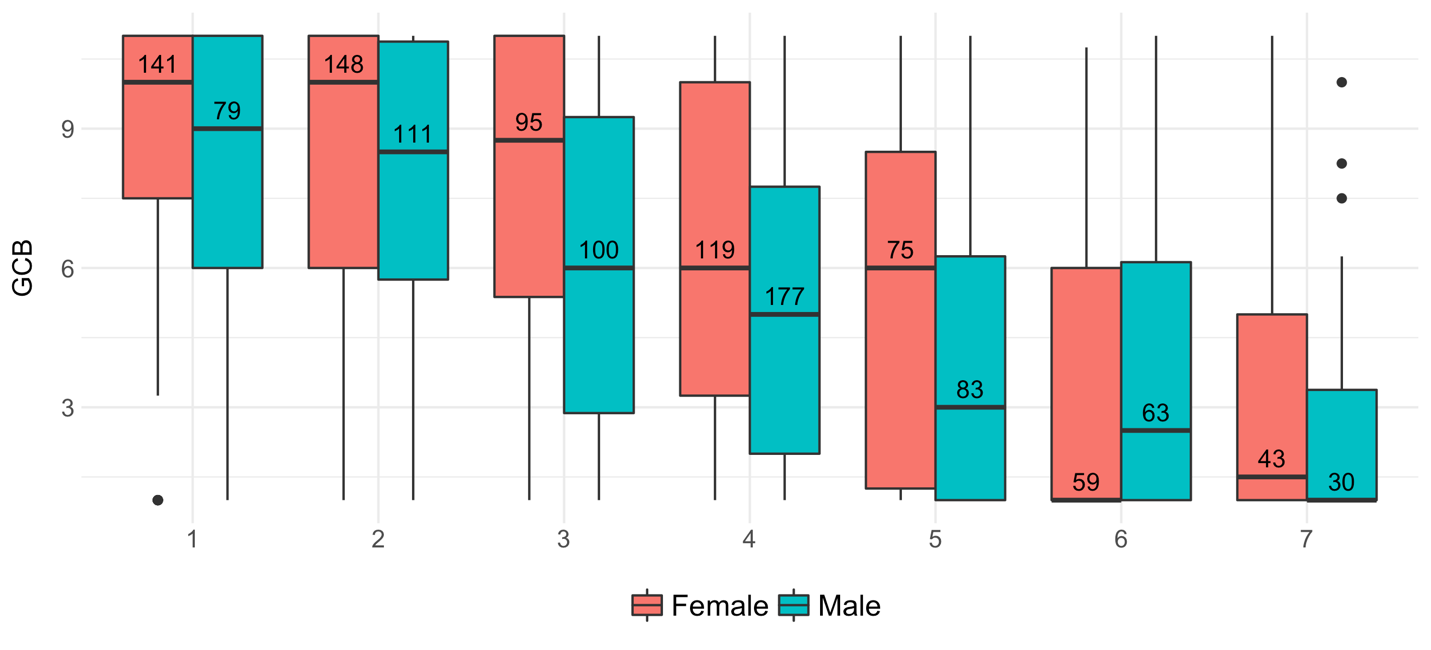


We created a dichotomous political ideology variable: ‘liberal’ = 1-3 (N=674) and ‘conservative’ = 4-7 (N=649). We then tested a model, with GCB as response variable, and participant sex X dichotomous political ideology as predictor. As seen in the table below, both main effects are significant but the interaction is not (*F_1,1319_* = 3.735, *P* = 0.0534). Post hoc contrasts showed that while conservative male and females did not differ significantly in GCB (*M_diff_* = 5.4, 95%CI[-12.26, 14.08], *P* = 0.1731), liberals male were lower on GCB than females (*M_diff_*  = 12.6, 95%CI[5.8, 19.38], *P* = 0.000009). Figure 5 shows this result.

*GCB ~ participant gender * political ideology.dichotomous*

| **Effect** | **DFn** | **DFd** | **F** | **p** | **ges** | **p<.05** |
| --- | --- | --- | --- | --- | --- | --- |
| **political.ideology** | **1** | **1319** | **267.5702** | **<0.0001** | **0.1686** | ***** |
| **participant.sex** | **1** | **1319** | **23.6666** | **<0.0001** | **0.0176** | ***** |
| political.ideology:participant.sex | 1 | 1319 | 3.7352 | 0.0535 | 0.0028 |  |

Figure 5. GCB as function of dichotomous political ideology variable, split by participant gender.


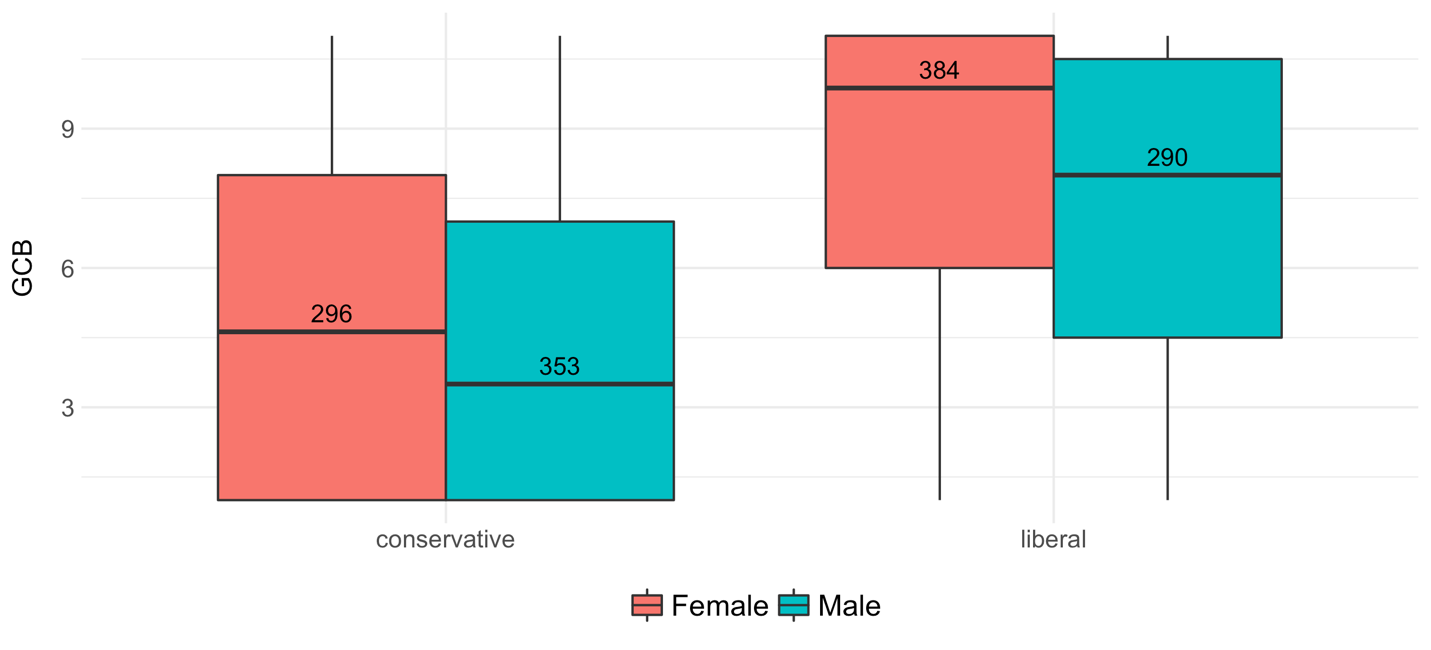


Figure 6. Post hoc pairwise comparison with Bonferroni correction (*Participant sex * Ideology*).

*
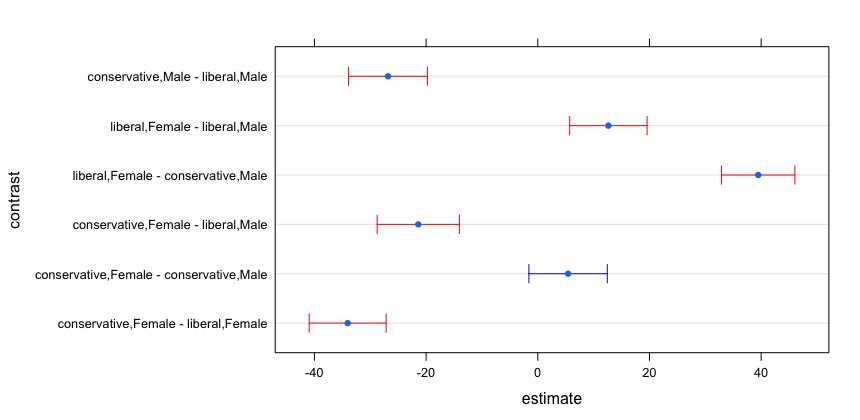
*

Gender difference by Ideology (Post Hoc with Bonferroni comparison):

|  | Ideology | estimate | SE | df | t.ratio | p.value |
| --- | --- | --- | --- | --- | --- | --- |
| Female-Male | conservative | 5.426293 | 2.657106 | 1319 | 2.042 | 0.0413 |
|  | liberal | 12.642196 | 2.62292 | 1319 | 4.82 | <.0001 |

### 4.3 Religion

We adopted the same strategy with religious identification, comparing people who identified with a religion (N = 695) with those who explicitly identified as Atheist/Agnostic (N = 516), leaving aside 100 subjects who picked the category ‘Other’ (e.g., spiritual, pagan, Wiccan, etc.). Similar to political ideology, religion explains significant variability in GCB, with non-religious people scoring higher on GCB than religious subjects. This relationship is not moderated by participant sex (*F* = 2.3157, *P* = 0.09) but post hoc contrasts with Bonferroni correction show that male and female participant differed more if they were Atheist/Agnostics (*M_diff_* = 19.23, 95%CI[11.07, 27.40], *P* < 0.0001) then if they identified with a religion (*M_diff_* = 11.40, 95%CI[4.40, 18.41], *P* = 0.00017). Figure 6 shows these results.

*GCB ~ participant gender * religion*

| **Effect** | **DFn** | **DFd** | **F** | **p** | **ges** | **p<.05** |
| --- | --- | --- | --- | --- | --- | --- |
| **participant.sex** | **1** | **1207** | **77.6993** | **<0.0001** | **0.0604** | **1** |
| **religion.dichotomous** | **1** | **1207** | **50.7985** | **<0.0001** | **0.0403** | **1** |
| participant.sex:religion.dichotomous | 1 | 1207 | 3.50574 | 0.0614 | 0.0028 | 1 |

Figure 6. GCB as function religious belief, split by participant sex.
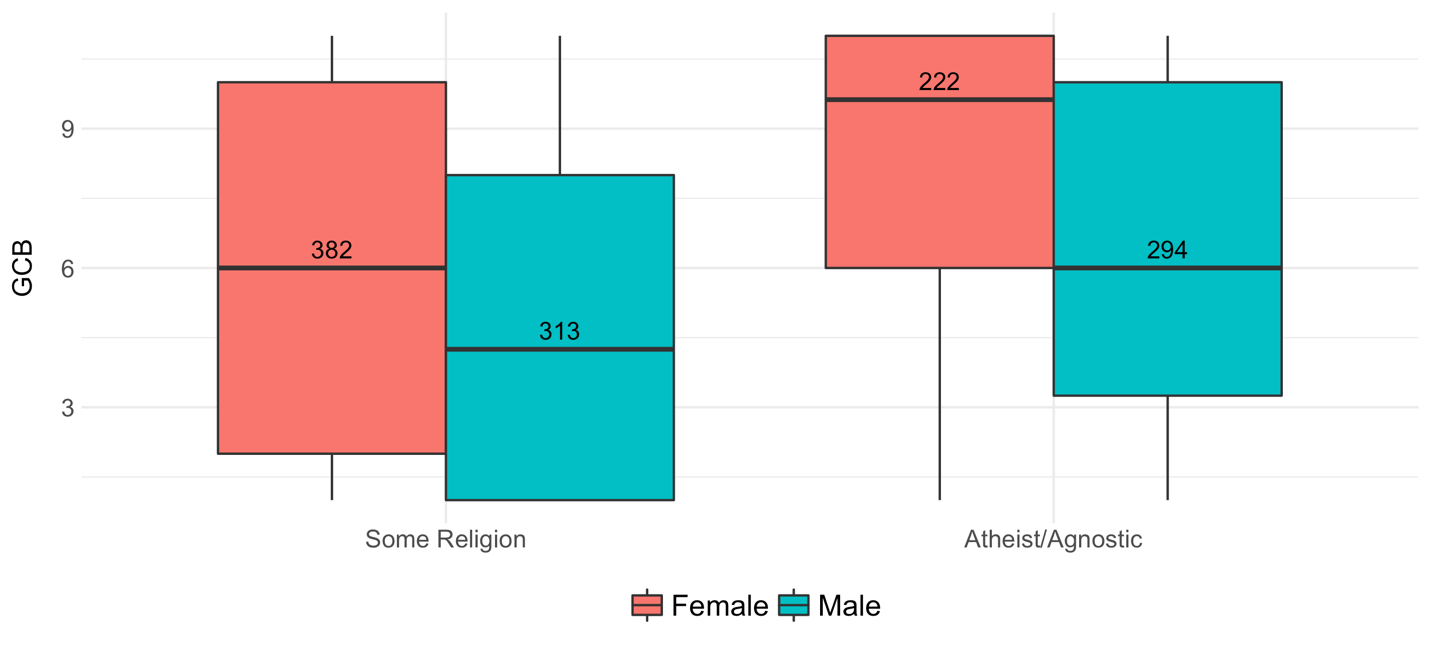


### 4.4 Race

Seventy five percent of our sample is White. However, while Whites (N = 999) and Non-whites (N = 324) do not differ in GCB, in Whites we found male and female to differ significantly in GCB (*M_diff_* = 13.92, 95%CI[7.89, 19.15], *P* < 0.0001) but not in Non-whites (*M_diff_* = 8.19, 95%CI[-18.78, 2.39], *P* = 0.1917). This sample is too imbalanced to warrant a conclusion. Figure 7 summarizes this result.

*GCB ~ participant gender * race.dichotomous*

| **Effect** | **DFn** | **DFd** | **F** | **p** | **ges** | **p<.05** |
| --- | --- | --- | --- | --- | --- | --- |
| race.new | 1 | 1319 | 0.1062 | 0.7445 | 0.0008 |  |
| **participant.sex** | **1** | **1319** | **37.7604** | **<0.0001** | **0.0278** | ***** |
| race.new:participant.sex | 1 | 1319 | 1.4591 | 0.2272 | 0.0011 |  |

Figure 7. GCB as a function of race, split by participant sex. *
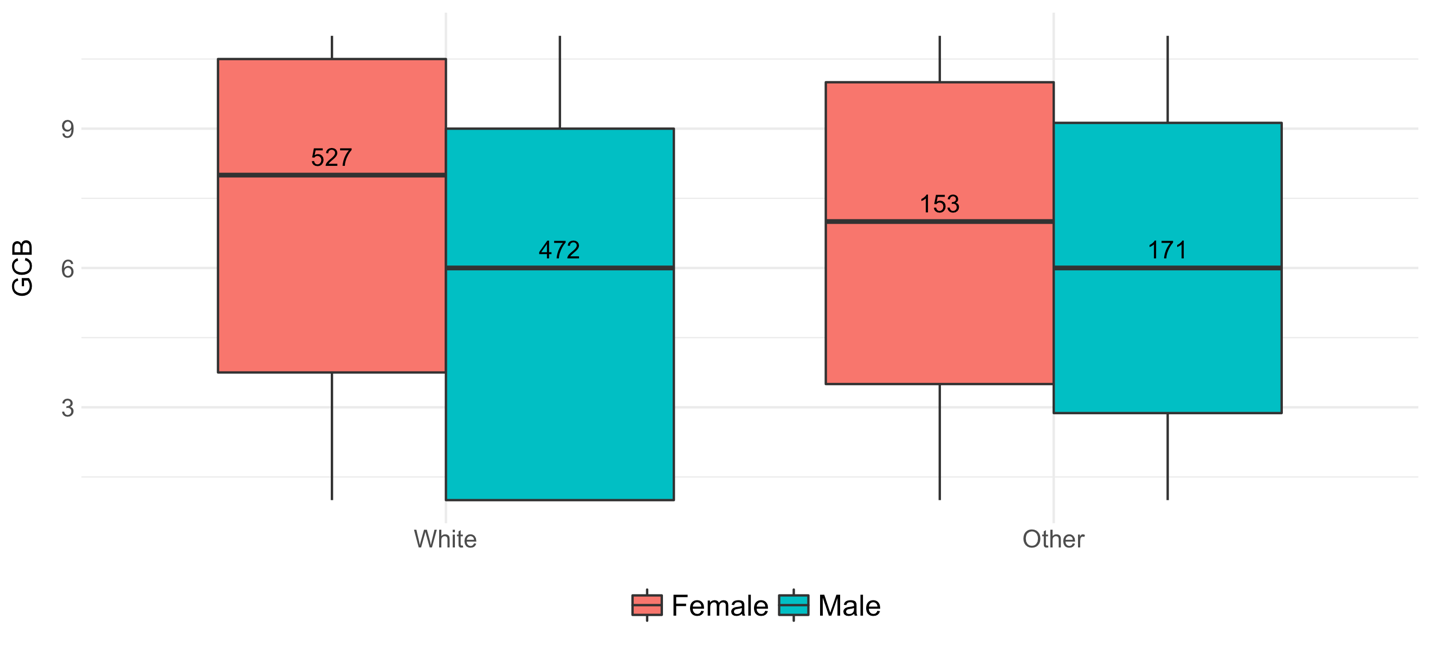
*

### 4.5 Geographical Region

We categorized the states where participants resided into 4 groups as follows:

- **Midwest**: Illinois, Indiana, Iowa, Kansas, Michigan, Minnesota, Missouri, North South Dakota, Nebraska, Ohio, Wisconsin
- **South:** Alabama, Florida, Georgia, Kentucky, Mississippi, North Carolina, South Carolina, Tennessee, Maryland, Virginia, West Virginia, Oklahoma
- **Northeast**: Maine, New York, New Jersey, Vermont, Massachusetts, Rhode Island, Connecticut, New Hampshire, Pennsylvania
- **West**: Alaska, Arizona, California, Colorado, Hawaii, Idaho, Montana, Nevada, New Mexico, Oregon, Utah, Washington, Wyoming

*GCB ~ region * participant gender*

| **Effect** | **DFn** | **DFd** | **F** | **p** | **ges** | **p<.05** |
| --- | --- | --- | --- | --- | --- | --- |
| **region** | **3** | **1313** | **3.5831** | **0.0013** | **0.0081** | ***** |
| **participant.sex** | **1** | **1313** | **40.4589** | **<0.0001** | **0.0298** | ***** |
| region:participant.sex | 3 | 1313 | 0.9037 | 0.439 | 0.0020 |  |

Post hoc tests show that gender difference in GCB is significant in the Northeast (*M_diff_*= 14.33, 95%CI[4.53, 28.22], *P* = 0.0372), the South (M_diff_ = 12.78, 95%CI[23.02, 25.36], *P* = 0.0039), and the West (*M_diff_* = 17.25, 95%CI[4.20, 30.31], *P* = 0.0016), but not the Midwest (*M_diff_* = 7.41, 95%CI[5.83, 20.64], *P* = 0.6872). Figure 8 shows these results.

Figure 8. GCB as function region, split by participant sex.


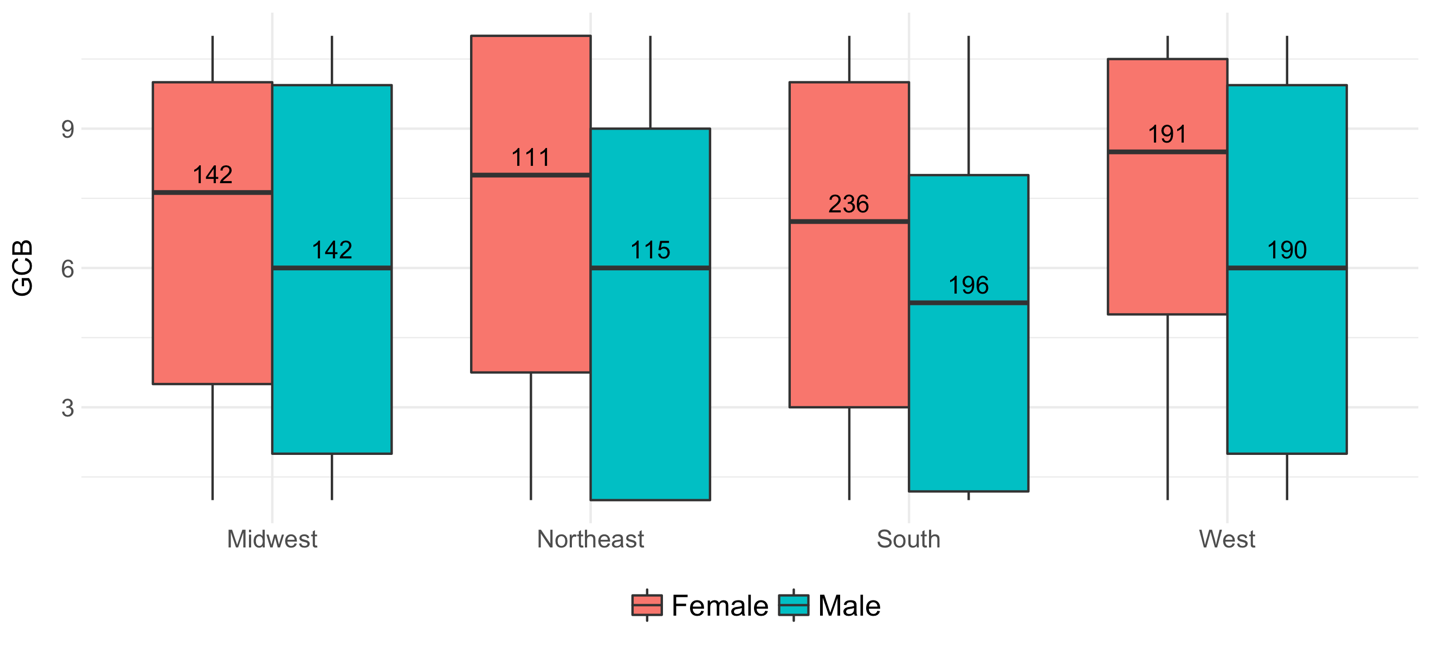


### 4.6 Combined Demographics

Finally, we combined all of the variables above (age, dichotomous political ideology, dichotomous religion, dichotomous race, and region) into a single composite score where a Midwestern, Religious, Conservative, White person would get a score of 4, and people missing any of those indices would get have a point subtracted (i.e., 0 meant Anywhere but Midwest, Atheist/Agnostic, Non-white, Liberal). This score summarizes the above breakdowns and is distributed as follows: 0 (5%), 1 (27%), 2 (35%), 3 (25%), 4 (%6).

####

*GCB ~ participant gender * composite score*

| **Effect** | **DFn** | **DFd** | **F** | **p** | **ges** | **p<.05** |
| --- | --- | --- | --- | --- | --- | --- |
| **composite score** | **4** | **1313** | **38.8976** | **<0.0001** | **0.10594** | ***** |
| **participant.sex** | **1** | **1313** | **42.1377** | **<0.0001** | **0.0310** | ***** |
| Participant.sex:composite score | 4 | 1313 | 0.3899 | 0.816 | 0.0011 |  |

###

Post hoc tests with Bonferroni correction showed that participants scoring 0 or 1 and participant scoring 3 and 4 on this composite score did not differ in GCB. Figure 9 show the mean differences with confidence intervals.

Figure 9. Mean differences in GCB as function of participant composite score and sex.


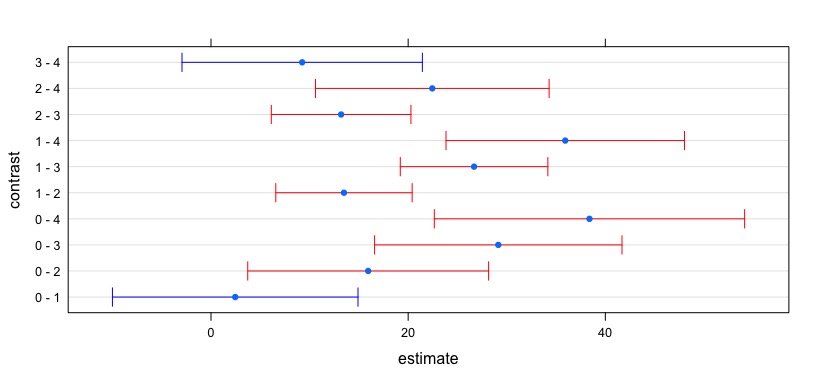


Figure 10. Distribution of sample over the composite score, plotted against GCB score, split by participant sex.


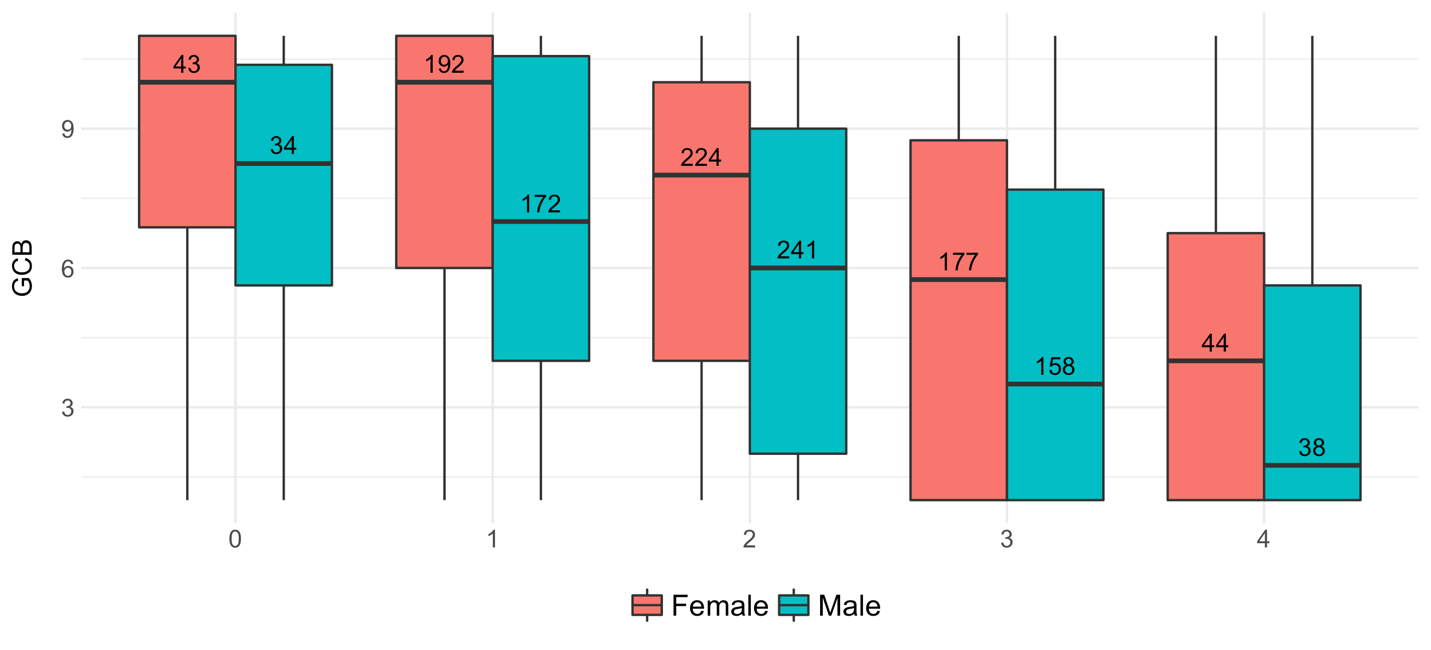


Male and female participants do not differ significantly in GCB on the two ends of the spectrum:
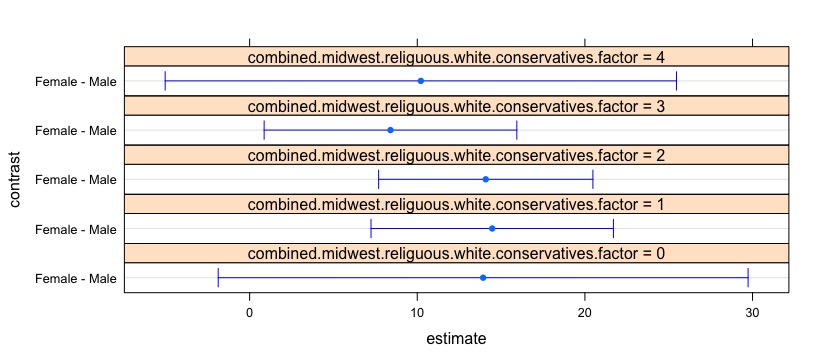


## E. Feelings toward Transgender People

In both studies, the perceived gender rating tasks were followed by feeling thermometer questions adapted from (Norton & Herek, 2013)^[[1]](#footnote-1)^:

*‘Using this scale from 0 to 100, please tell us your personal feelings toward each of the following groups. As you do this task, think of an imaginary thermometer. The warmer or more favorable you feel toward the group, the higher the number you should give it. The colder or less favorable you feel toward the group, the lower the number. If you feel neither warm nor cold toward the group, rate it 50: [*Men, Women, Trans men, Trans women*].’*

Feeling thermometers for trans men and trans women were correlated (Study 1: Spearman $\rho$= 0.954 *P* < 0.0001; Study 2: Spearman $\rho$= 0.925, *P* < 0.0001). They were averaged into a feeling toward trans people score. Figure 11 shows the distribution of this score for each target.

Figure 11. Density plots for feeling thermometer scores, split by study, target, participant gender.
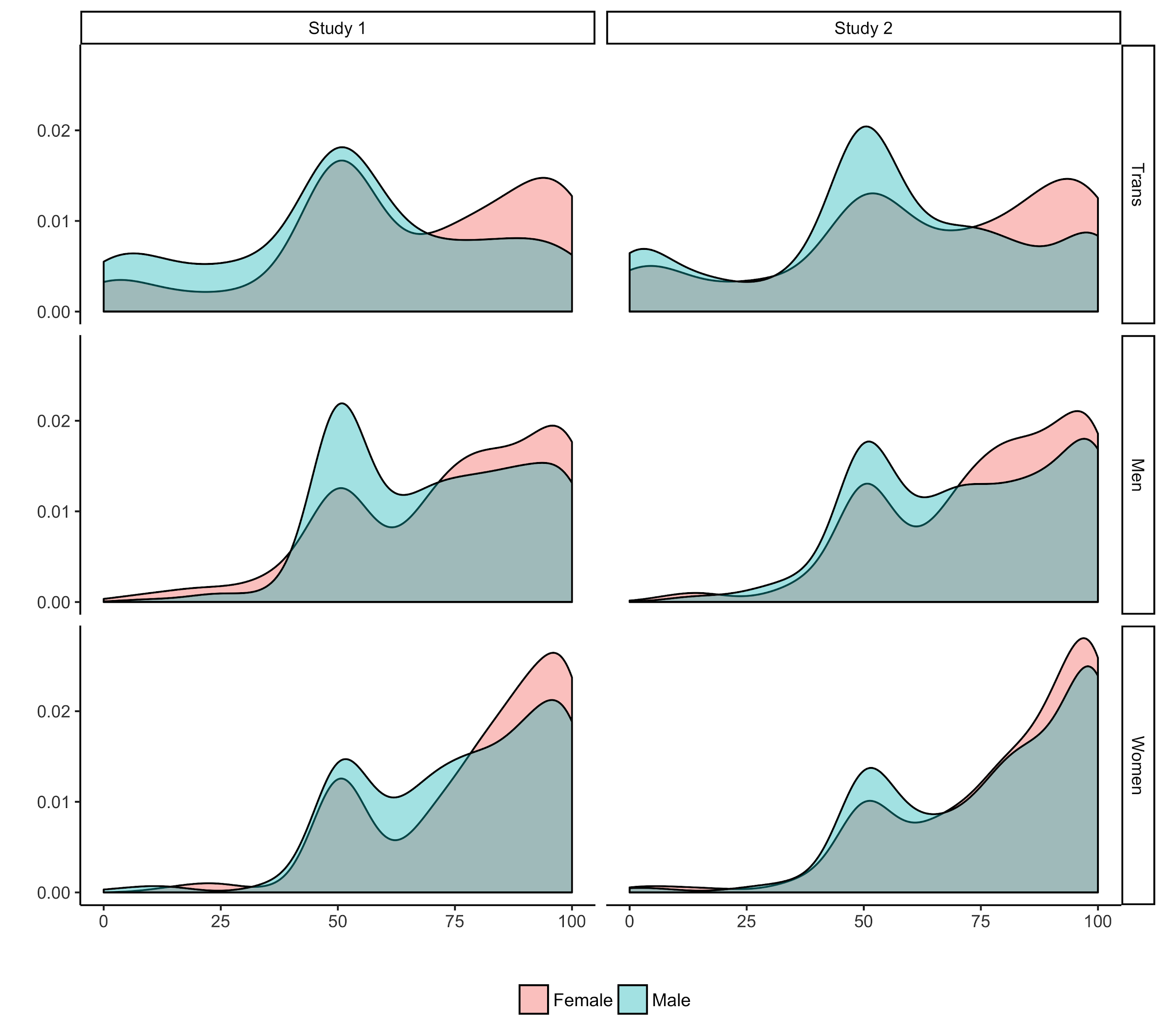


Table below shows feeling thermometers by study, participant gender and target.

| study | participant.sex | variable | n | mean | sd | min | max | range | se |
| --- | --- | --- | --- | --- | --- | --- | --- | --- | --- |
| 1 | Female | **Transgender** | **329** | **65.2781** | **27.8376** | **0** | **100** | **100** | **1.5347** |
|  |  | Men | 329 | 74.5683 | 22.1311 | 3 | 100 | 97 | 1.2201 |
|  |  | Women | 329 | 79.9848 | 19.4844 | 13 | 100 | 87 | 1.0742 |
|  | Male | **Transgender** | **294** | **52.8180** | **28.4209** | **0** | **100** | **100** | **1.6575** |
|  |  | Men | 294 | 70.8707 | 19.8962 | 9 | 100 | 91 | 1.1603 |
|  |  | Women | 294 | 76.2993 | 19.9209 | 2 | 100 | 98 | 1.1618 |
| 2 | Female | **Transgender** | **351** | **63.3618** | **30.3309** | **0** | **100** | **100** | **1.6189** |
|  |  | Men | 351 | 76.1168 | 20.4446 | 7 | 100 | 93 | 1.0912 |
|  |  | Women | 351 | 80.3675 | 20.4205 | 1 | 100 | 99 | 1.0899 |
|  | Male | **Transgender** | **349** | **55.0444** | **28.7808** | **0** | **100** | **100** | **1.5406** |
|  |  | Men | 349 | 72.7249 | 21.0607 | 12 | 100 | 88 | 1.1273 |
|  |  | Women | 349 | 78.3409 | 20.8218 | 0 | 100 | 100 | 1.1145 |

####

### 5.1 Feelings and GCB

In both studies, values for feeling thermometers when the target was ‘trans man’ or ‘trans women’ were correlated with GCB scores, but not when the target was ‘Men’ or ‘Women’ (Table below).

Spearman correlations between feeling thermometers and GCB, split by target and participant sex (****: *P* < 0.0001)

| Participant sex | Male | | | Female | | |
| --- | --- | --- | --- | --- | --- | --- |
| Target | Transgender | Women | Men | Transgender | Women | Men |
| Study 1 | **0.44****** | -0.12* | -0.09 | **0.42****** | -0.01 | -0.07 |
| Study 2 | **0.53****** | 0.04 | 0.02 | **0.58****** | 0.06 | -0.04 |

Figure 12. GCB plotted against feelings of warmth toward Men, Women, and Transgender people for Study 1 & Study 2, using LOESS (span =0.65).


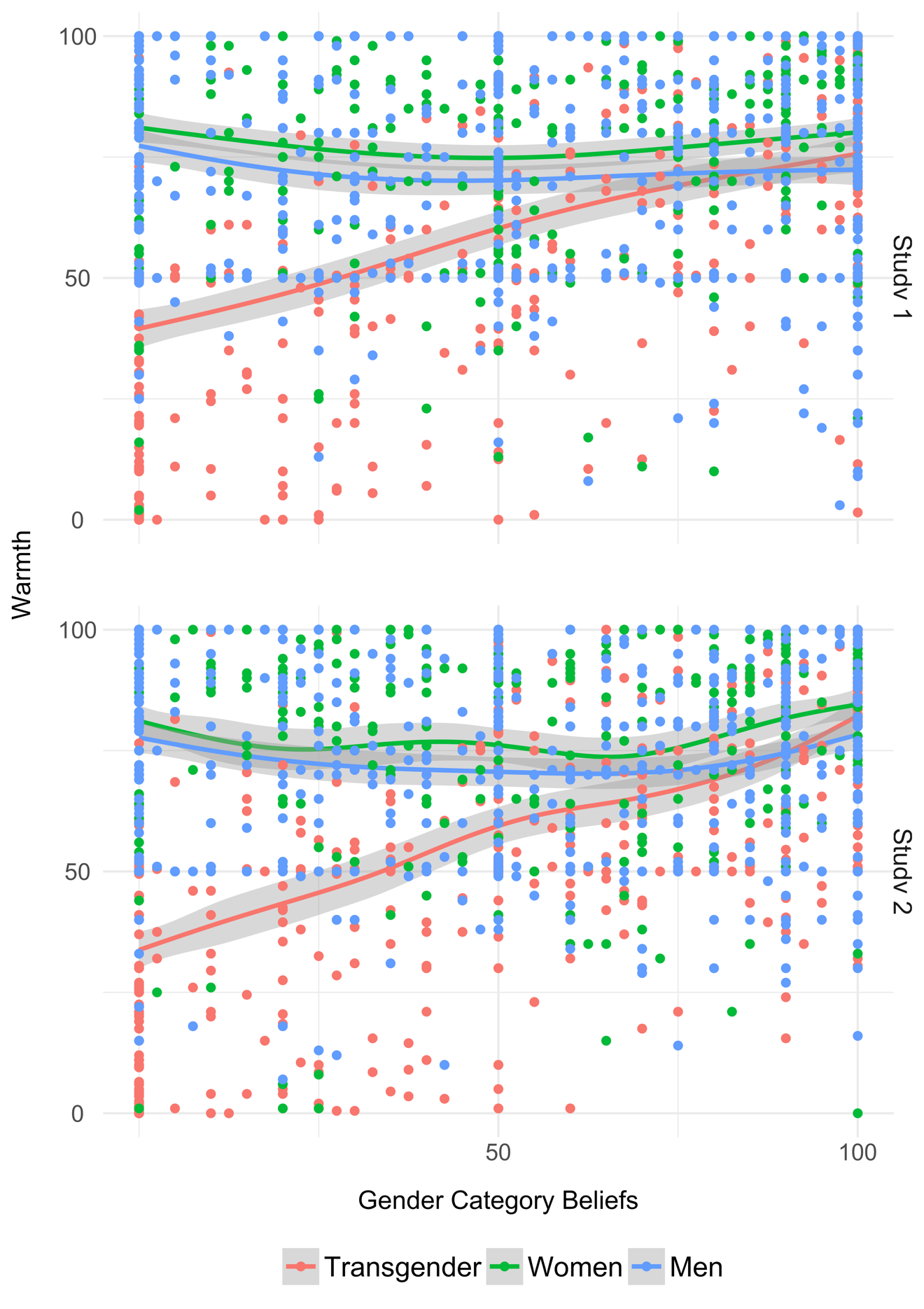


## F. Bathroom Choice Responses

All participants were asked the question below about transgender bathroom policy:

‘*It would be wrong to allow a transgender man [woman] (a person who identifies as a man [woman] but was designated female [male] at birth) to use the men's restroom.*’

They could select one of two choices, ‘*Yes, it is wrong*’ or ‘*No, it is not wrong*’, coded as 0 and 1. Table below shows distribution of responses by male and female participants, for both Study 1 and Study 2.

Percentage of participants thing it is wrong/non-wrong for trans men and trans women to use their bathroom of choice.

| Participant sex | | Female | | Male | |
| --- | --- | --- | --- | --- | --- |
| Bathroom choice for | | Trans women | Trans men | Trans women | Trans men |
| Study 1 | *Yes, it is wrong* | 13.6% | 13.0% | 16.3% | 14.6% |
|  | *No, it is not wrong* | 39.1% | 39.8% | 30.8% | 32.5% |
| Study 2 | *Yes, it is wrong* | 14.4% | 14.0% | 17.4% | 16.7% |
|  | *No, it is not wrong* | 35.7% | 36.1% | 32.4% | 33.1% |

Crosstabs below shows the degree of consistency in how participants responded to the two questions (i.e. about Trans men vs Trans women).

| Study 2 | | Trans men | |  | Study 2 | | Trans men | |
| --- | --- | --- | --- | --- | --- | --- | --- | --- |
|  |  | Wrong | Not wrong |  |  |  | Wrong | Not wrong |
| Trans women | Wrong | 26.7% | 3.1% |  | Trans women | Wrong | 29.0% | 2.6% |
|  | Not wrong | 0.7% | 69.2% |  |  | Not wrong | 1.3% | 66.8% |

### 6.1 Feelings vs. GCB – Logistic Regressions

Using only consistent responses, we tested and compared the following models to predict policy preferences.

*Transgender people’s right to use bathroom of choice (y/n) ~ GCB*

Tables below show the results for these logistic regression models in the two studies:

| Study 1 | Estimate | Std.Error | Z value | Pr(>\|z\|) |  |
| --- | --- | --- | --- | --- | --- |
| (Intercept) | -0.68519 | 0.15611 | -4.389 | 0.0000114 | *** |
| GCB | 0.03863 | 0.00342 | 11.294 | <2e-16 | *** |

Exponentiated coefficients:

Intercept: 0.5039, 95%CI[0.369, 0.682]

GCB: 1.0393, 95%CI[1.032, 1.046]

| Study 2 | Estimate | Std.Error | Z value | Pr(>\|z\|) |  |
| --- | --- | --- | --- | --- | --- |
| (Intercept) | -1.246177 | 0.162958 | -7.647 | 2.05E-14 | *** |
| GCB | 0.049355 | 0.003699 | 13.344 | <2e-16 | *** |

Exponentiated coefficients:

Intercept: 0.287, 95%CI[0.207, 0.393]

GCB: 1.050, 95%CI[1.043, 1.058]

In each case, we compared the model above to one with feeling thermometer scores as predictor:

*Transgender people’s right to use bathroom of choice (y/n) ~ feeling thermometers*

Both variables explained significant variance in responses to the bathroom choice question; however, GCB captured a larger proportion of variance.

##

Study 1:

| model | LogLik | Chisq | Pr(>Chisq) | McFadden R^2^ |
| --- | --- | --- | --- | --- |
| trans.bathroom ~ 1 | 354.17 |  |  |  |
| trans.bathroom ~ GCB | 262.37 | 183.6 | < 2.2e-16 *** | 25.91% |
| trans.bathroom ~ trans.feeling.thermometers | 290.10 | 128.15 | < 2.2e-16 *** | 18.09% |

Study 2:

| model | LogLik | Chisq | Pr(>Chisq) | McFadden R^2^ |
| --- | --- | --- | --- | --- |
| trans.bathroom ~ 1 | 413.34 |  |  | 0 |
| trans.bathroom ~ GCB | 261.95 | 302.78 | < 2.2e-16 *** | 36.62% |
| trans.bathroom ~ trans.feeling.thermometers | 275.73 | 275.22 | < 2.2e-16 *** | 33.29% |

### 6.2 Feelings vs. GCB – Cross Validation

We report how three models with GCB, Warmth, or GCB + Warmth perform in predicting bathroom choice questions. The GCB score however, are campsites of 4 items whereas the Warmth scores are campsites of 2. It is possible that the GCB is simply better at predicting bathroom choice responses because it is a more reliable score.

Here we compare the 4 additional models:

1. GCB.male/female
2. GCB.man/women
3. GCB.male/female + Warmth
4. GCB-man/women + Warmth

Table below shows the mean AUC of each model using 10-fold cross validation.

|  | model | n | mean | sd | min | max | range | se |
| --- | --- | --- | --- | --- | --- | --- | --- | --- |
| 1 | GCB.man/women+thermometers | 10 | 0.892 | 0.017 | 0.868 | 0.93 | 0.062 | 0.005 |
| 2 | GCB+thermometers | 10 | 0.892 | 0.017 | 0.868 | 0.93 | 0.062 | 0.005 |
| 3 | GCB | 10 | 0.858 | 0.033 | 0.812 | 0.92 | 0.108 | 0.01 |
| 4 | GCB.man/women | 10 | 0.853 | 0.033 | 0.803 | 0.915 | 0.113 | 0.01 |
| 5 | GCB.male/female | 10 | 0.839 | 0.032 | 0.793 | 0.904 | 0.111 | 0.01 |
| 6 | GCB.male/female+thermometers | 10 | 0.818 | 0.029 | 0.747 | 0.849 | 0.102 | 0.009 |
| 7 | theremometers | 10 | 0.818 | 0.029 | 0.747 | 0.849 | 0.102 | 0.009 |

The two GCB ratings (man/woman and male/female) perform better than thermometer ratings. The best models are those that use both GCB and thermometers ratings. The figure below plots the AUCs of different folds, and the average AUCs.


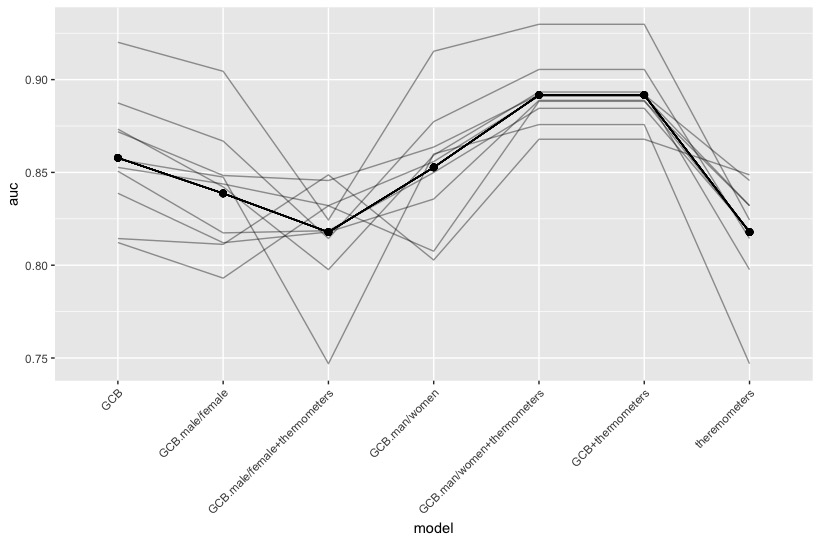


## G. Additional Analyses

The two tables below show the frequency with which participants chose ends of the scale in the gender transformation task (0% or 100%), for the combined sample from the two studies and split by Frame and participant gender (Note that these include up to 4 responses from each participant):

| 0% | *identified gender* | *assigned gender* |  | 100% | *identified gender* | *assigned gender* |
| --- | --- | --- | --- | --- | --- | --- |
| Female | 298  (21.9%) | **414**  **(30.5%)** |  | Female | **382**  **(29.6%)** | 294  (22.8%) |
| Male | **394**  **(29%)** | 250  (18.4%) |  | Male | 238  (18.4%) | **374**  **(29%)** |

$\chi$(1) = 49.771, *P* < 0.0001 $\chi$(1) = 39.242, *P* < 0.0001

Female participants were more likely to choose ‘*0%*’ in frames asking about the assigned gender than in frames asking about the identified gender (21.5% vs. 29.8%) yet male participants were more likely to choose 0% in frames asking about the self-identified gender than in frames asking about the assigned gender (31.4% vs. 19.9%). For the choice ‘*100%*’ the reverse was true. While a larger proportion of female participants chose 100% in frames asking about the self-identified gender than in frames asking about the assigned gender (27.5% vs. 21.2%), male participant chose 100% in frames asking about the assigned gender than in frames asking about the self-identified (18.9% vs. 29.8%).

Coding the two extremes of GCB as 0, and 1, and 0.5 for anyone in between, gives the following distribution:

| GCB | Female | Male |
| --- | --- | --- |
| 0 | 114 (8.6%) | 157 (11.8%) |
| 0.5 | 413 (31.2%) | 394 (29.7%) |
| 1 | 153 (11.5%) | 92 (6.9%) |

We tested the model below to predict affect scores: *Thermometers ~ Participant Sex * GCB.trichotomized*

|  | Df | Sum Sq | Mean Sq | F value | P-value |  |
| --- | --- | --- | --- | --- | --- | --- |
| Participant.sex | 1 | 34807 | 34807 | 52.6716 | 6.72E-13 | *** |
| GCB.trichotomized | 2 | 224664 | 112332 | 169.9847 | <2.20E-16 | *** |
| Participant.sex * GCB.trichotomized | 2 | 7531 | 3765 | 5.6978 | 0.003436 | ** |

Pairwise comparison with Bonferroni correction showed that on the two ends of the GCB scale, male and female subjects do not differ in affect towards transgender people. However, in those with mixed GCB, female subjects report higher positive affect than males toward transgender people. The plot below shows these contrasts.


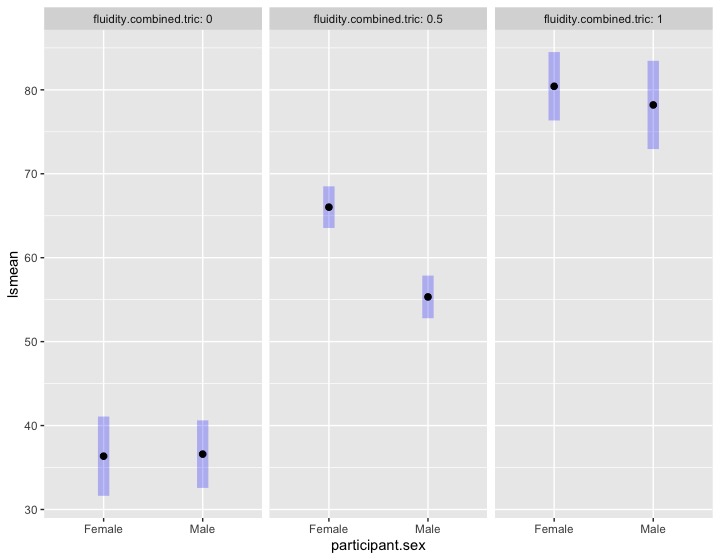


## H. Open-ended Responses

###

The gender transformation task included a scale and an open-ended probe, ‘*Can you explain your reasoning?*’ (see section 1.2). This item allowed us to ensure that participants spent a few moments thinking and expressing their thoughts about the scenarios, and could adjust their choices on the scale accordingly. In a sense, this item served as a comprehension check.

We examined and coded the contents of these explanations into three broad categories. First category was Biology, which was present in almost half of the explanations (44.5%), This category includes mentions of *body parts*, *genes*, *anatomy*, *DNA*, *penis, vagina*, *reproduction*, *hysterectomy*, *chromosomes*, etc. Explanations in this category ranged from transition between genders is ‘*not possible at all*’ to ‘*almost full yet incomplete*’. Some examples of this type of explanations are,

- *Sex is something biological, not a mindset. Just as I am a white male, I would not be a black female just because I thought I was.*
- *A woman is born not made.*
- *She has a vagina, making her a woman.*
- *Its a fake vagina so no not a woman.*
- *Jill was born female. She will always be female. She might like "manly" things and associate more as a man, but at the end of the day, she is still a female.*
- *She was born with a vagina, that is what makes her a female...period...no if ands or buts.*
- *100% woman... whatever is in her head is a lie and she needs a therapy to help her accept who she is*

Another category was ‘Choice’, where purely biological basis of gender was rejected and self-identification was treated as the necessary and sufficient criteria to determine someone’s gender. Examples of this type of explanation include:

- *Patrick is male. No matter what he his gender was at birth, he identifies as a male... PERIOD, END OF STORY!*
- *Patrick is 100% male and 0% female, since gender is not determined by sexual characteristics or upbringing.*
- *You are who you feel like you want to be.*
- *Patricia presents and acts as a woman, with name, clothes, lipstick, and possibly other behaviors. Therefore, as far as I am concerned, she is a woman.*
- *Your gender at birth does not determine your gender, YOU define that for yourself.*
- *Transwomen are women. If that is how she thinks of herself, then that is what she is.*
- *Surgery, counseling, and hormone therapy make him a man.*

We call a third category was ‘Mixed’ where self-identification and biology were both present:

- *Jack identifies as a man and should be seen as such. I only say 90% because he is still technically genetically female*
- *I'm an atheist and have no religious motivation for answering this way, but still believe there are immutable characteristics associated with biological sex. That being said, I would strive to treat this person as 100% the gender of their choice should I meet them, but doing so would not necessarily be an accurate reflection of my inner thoughts.*
- *Underlying physical characteristics count for something, but if his overwhelming identity is as a man, I support that.*
- *This is tough. Since she was born a woman, I don't think we can just ignore that and throw it out completely.*
- *Patrick is choosing to live as a male, so even though she still has female characteristics, I believe she is just as much male as female.*
- *I felt this due to Patricia still physically being a man. I understand that they feel and relate more to being feminine however they still have the bone structure, sex organ and strength of a man. I respect their mindset so I just pushed them over the 50 percent mark as a man due to the body itself with respect to their feeling as if they are a woman. Say someone relates to being an animal, they may mentally believe they are a cat and behave that way and maybe are accepted by others for the behaviors but they are still human.*
- *This person has taken all the steps necessary to transform them self into a woman, at least physiologically. The one step remaining, that they can never achieve, is the ability to conceive and bear children. So outwardly this person will be female but never completely so; they will be in the limbo between sexes.*

Across conditions, similar proportions of explanations were coded under ‘Biology’, ranging between 43% to 49% (See table below). A lower proportion of explanations was coded as ‘Mixed’ in the Non-biological condition (20%-26%), and ‘Choice’ in the Biological condition (20%-26%). This pattern did not differ depending on the direction of transformation.

|  | | Biology | Mixed | Choice |
| --- | --- | --- | --- | --- |
| Jack.to.Jill | Biological | 0.430 | 0.363 | **0.206** |
|  | Non-biological | 0.491 | **0.208** | 0.300 |
| Jill.to.Jack | Biological | 0.462 | 0.271 | **0.266** |
|  | Non-biological | 0.452 | **0.256** | 0.290 |

Participant explanations coded under ‘Biology’ did not differ depending on participant gender (See table below). However, explanations by female participants were more likely to be coded under ‘Choice’ than male participants (31% vs. 23%). Conversely, explanations from male participants were more frequently coded under the category ‘Mixed’ than those of female participants (28% vs 23%).

|  | | Biology | Mixed | Choice |
| --- | --- | --- | --- | --- |
| Participant gender | Female | 0.453 | 0.232 | 0.313 |
|  | Male | 0.484 | 0.285 | 0.229 |

We further examined explanations labelled as ‘Biology’ (see table below). Majority of responses in this category centered on how the gender one is born with stays the same and cannot be changed (40%). Examples of this type of reasoning were:

- *‘Gender is 100% biological. There are only 2 genders. Patrick was born as a male and will always be a male, regardless of how he feels.’*
- *‘In my opinion, your gender is determined at birth, regardless of how you attempt to modify your appearance.’*
- *‘She is somewhat like a man by the way she acts and appears, but she is still a woman because that is her birth gender.’*

| Biology based explanations | birth | 0.408 | hormones | 0.002 |
| --- | --- | --- | --- | --- |
|  | reproduction | 0.014 | socialization | 0.065 |
|  | genes/  chromosomes | 0.128 | organs | 0.361 |

Bodily organs were mentioned in 36% of ‘Biology’ explanations:

- *‘This man does not have female genitals.’*
- *‘If you are born with female anatomy, then you are a woman, regardless of whether you get elective surgery to try and change yourself.’*
- *‘Patrick is mostly male, with the exception of some bodily structures left over from his birth sex.’*

Chromosomes and genes were mentioned in 12% of explanations:

- *‘I am going to just go by his Y chromosome. No amount of surgery is going to do away with this, IMO.’*
- *‘Can not change your DNA.’*

Upbringing and experience living as one gender was mentioned in around 5% or responses:

- *‘She is undergoing changes to be more male, so her hormones are more male and she will have some physical characteristics of a male but still must have some female characteristics since she was raised this way.*
- *‘This person chooses to present as a woman and as such primarily thinks of themselves as a woman but lacks the entirety of a woman's experience do to the lack of medical/hormonal intervention.’*

Male or female participants, or participants assigned to Biological or Non-biological transformation conditions did not differ in using these subcategories in the explanations. See table below.

|  | Participant sex | |  |  | Transformation type | |
| --- | --- | --- | --- | --- | --- | --- |
|  | Female | Male |  |  | Biological | Non-biological |
| birth | 0.401 | 0.435 |  |  | 0.453 | 0.435 |
| reproduction | 0.013 | 0.019 |  |  | 0.026 | 0.006 |
| genes | 0.144 | 0.096 |  |  | 0.144 | 0.134 |
| hormones | 0.006 | 0 |  |  | 0.006 | 0.006 |
| socialization | 0.052 | 0.070 |  |  | 0.006 | 0 |
| organs | 0.368 | 0.358 |  |  | 0.078 | 0.064 |

1. [A. T. Norton, G. M. Herek, Heterosexuals’ attitudes toward transgender people: Findings from a national probability sample of US adults. *Sex Roles*. **68**, 738–753 (2013).](http://paperpile.com/b/x7bKFB/LvI6) [↑](#footnote-ref-1)
